# Enhanced beetle antennae search algorithm for complex and unbiased optimization

**DOI:** 10.1007/s00500-022-07388-y

**Published:** 2022-08-21

**Authors:** Qian Qian, Yi Deng, Hui Sun, Jiawen Pan, Jibin Yin, Yong Feng, Yunfa Fu, Yingna Li

**Affiliations:** 1 Yunnan Key Laboratory of Computer Technology Applications, Faculty of Information Engineering and Automation, Kunming University of Science and Technology, Kunming, 650500 China; 2 Faculty of Foreign Languages and Cultures, Kunming University of Science and Technology, Kunming, 650500 China

**Keywords:** Meta-heuristic algorithm, Beetle antennae search (BAS), Adaptive step size reduction, Contemporary optimal update, Multi-directional sensing

## Abstract

Beetle Antennae Search algorithm is a kind of intelligent optimization algorithms, which has the advantages of few parameters and simplicity. However, due to its inherent limitations, BAS has poor performance in complex optimization problems. The existing improvements of BAS are mainly based on the utilization of multiple beetles or combining BAS with other algorithms. The present study improves BAS from its origin and keeps the simplicity of the algorithm. First, an adaptive step size reduction method is used to increase the usability of the algorithm, which is based on an accurate factor and curvilinearly reduces the step size; second, the calculated information of fitness functions during each iteration are fully utilized with a contemporary optimal update strategy to promote the optimization processes; third, the theoretical analysis of the multi-directional sensing method is conducted and utilized to further improve the efficiency of the algorithm. Finally, the proposed Enhanced Beetle Antennae Search algorithm is compared with many other algorithms based on unbiased test functions. The test functions are unbiased when their solution space does not contain simple patterns, which may be used to facilitate the searching processes. As a result, EBAS outperformed BAS with at least 1 orders of magnitude difference. The performance of EBAS was even better than several state-of-the-art swarm-based algorithms, such as Slime Mold Algorithm and Grey Wolf Optimization, with similar running times. In addition, a WSN coverage optimization problem is tested to demonstrate the applicability of EBAS on real-world optimizations.

## Introduction

This section will introduce the background and the motivation of the research.

### Research Background

Based on empirical data, functional models can be built to summarize the key characteristics of the data and to predict the tendency of new data, thus helping to promote the social productivity. The goodness of a functional model is not only based on its structure (such as number of parameters and model capacity), but also related to the utilized parameter optimization algorithms. If the optimization algorithm cannot find optimal parameters, the advantages of the model will be diminished. The traditional parameter optimization algorithms, such as the gradient method, rely on the use of mathematical derivation, which is usually impossible for complex models. Even the most famous gradient descent method (Bertsekas 1999; Langford et al. 2009; Tseng and Yun 2009) is vulnerable to gradient disappearance or gradient explosion problems. In solving the problem of complex functional optimizations, meta-heuristic intelligent optimization algorithms that do not rely on mathematical derivation play an increasingly important role. The most well-known meta-heuristic algorithm is the genetic algorithm (GA), which imitates the process of biological evolution in nature and uses selection, crossover, and mutation operations to iteratively find the optimal solution (Holland 1992). The advantage of this type of algorithm is that it does not depend on the specific problem, and it is applicable as long as the model parameters can be represented as a solution in the solution space and a fitness function can be provided to evaluate the goodness of the solution. In addition to GA, optimization algorithms that simulate biological behavior or natural phenomena include Particle Swarm Optimization (PSO) (Kennedy and Eberhart 1995; Ghasemi et al. 2019), Simulated Annealing (SA) (van Laarhoven and Aarts 1987), Slime Mold Algorithm (SMA) (Li et al. 2020; Naik et al. 2021), Harris Hawk Optimization (HHO) (Heidari et al. 2019; Fan et al. 2020), Sparrow Search Algorithm (SSA) (Xue and Shen 2020), Whale Optimization Algorithm (WOA) (Nasiri and Khiyabani 2018), Grey Wolf Optimization (GWO) (Kohli and Arora 2018), Flower Pollination Algorithm (FPA) (Yang 2012), Ant Colony Optimization (ACO) (Dorigo 1999), Artificial Bee Colony (ABC) (Karaboga and Basturk 2007), and so on. Among them, SMA is proposed based on the oscillation mode of slime mold in nature, SSA simulates the predation and anti-predation behavior of sparrow population, GWO imitates the predation behavior of grey wolf group. Including the above-mentioned algorithms, the most distinctive feature of many state-of-the-art algorithms is that a swarm of individuals are utilized to increase the searching efficiency. However, the cost of these swarm-based algorithms is the increased complexity and prolonged running time.

Beetle Antennae Search (BAS) is a relatively new bio-inspired intelligent optimization algorithm (Jiang and Li 2018; Zhang et al. 2021), which imitates the detecting and searching behavior of longhorn beetles. Different from the above-mentioned many swarm-based algorithms, BAS only simulated the behavior of one beetle, the origin of BAS is as follows. In nature, beetles rely on their two antennae to recognize the smell of potential food source or opposite sex in the space and are guided by the smell information to jump in its direction. BAS simulates the foraging process of the beetle and simplifies the beetle to a solution in a multi-dimensional solution space. Based on the current position of the beetle and a random direction, two new solutions (called left and right solutions), which represents the positions of the beetle antennae, can be calculated. And then, the beetle moves with a certain step size in the direction of the solution with the better fitness among the left and right solutions until the optimal solution is found or the maximum number of iterations is reached. The specific process of BAS can be found in Sect. 2.

BAS and its improved versions have already been used in many applications, such as ship collision avoidance (Xie et al. 2019) and path planning of mobile robots (Wu et al. 2020; Zhou et al. 2021). BAS has some advantages, such as few parameters and simplicity, mainly because only one beetle is utilized. As a result, the time complexity of BAS is only O (K * N), where K is the problem dimension, and N is the maximum number of iterations. In contrast, the time complexity of SMA is O (K + N * P *(1 + logP + K)), where P is the number of cells of slime mold. Because of its simplicity, BAS requires less running time and it is easy to be combined with other swarm-based algorithms as a kind of global or local search strategy. For example, Lin et al. (2018) combined BAS and PSO to form a new algorithm, which utilized the global search ability of PSO and the local search ability of BAS. Fan et al. (2021) proposed an improved GWO algorithm (BGWO) by giving the alpha wolf in the GWO algorithm the BAS-like ability. By this ability, alpha wolf can sense prey by left and right ears, thus better leading the wolves to the optimal solution. Shao et al. (2018) utilized BAS as an additional strategy after the global search formula of FPA, thus improving the convergence rate of algorithm. Zhao et al. (2020) proposed new improvement methods to BAS and GA, respectively, then combined these two algorithms to form BGA, which has better performance than the other two hybrid algorithms. Zhou et al. (2019) integrated BAS with the procedure of SA. At each temperature, SABAS searches solution space multiple times and the step size is proportional to the current temperature, thus improving the global and local search abilities of the algorithm. In a similar way, BAS has been combined with ACO or ABC to form BCO and MABC (Zhang et al. 2020a, b) algorithms, respectively. These studies proved the usability of BAS as an additional strategy to improve other intelligent algorithms.

### Improvement methods of BAS and motivation of current study

Although BAS has its own advantages, its optimization ability when used alone is unsatisfactory, especially for the complex and high-dimensional problems. One common way to increase the efficiency of BAS is to utilize the idea of swarm intelligent algorithms. In other words, multiple beetles, rather than one beetle, can be used to search solution space simultaneously. For example, Wang et al. (2018) extended BAS into Beetle Swarm Optimization algorithm (BSO) by increasing the number of beetles and utilizing the idea and formulas of PSO. Xu et al. (2020) proposed an algorithm called Beetle Antennae Search based on Levy flights and Adaptive strategy (LABAS). LABAS used a population of beetles and combined many searching strategies, such as Levy flights, an adaptive step size strategy, and a generalized opposition-based learning method. Zhao and Qian (2018) also used a population of beetles for searching and added the group experience into the position update formula of BAS, this new Learning and Competing Chaos Beetle Swarm Algorithm (LCCBSA) showed better performance than PSO and GA. These beetle swarm algorithms are indeed superior to original BAS. However, such improvement method inevitably increased the computational complexity of the algorithms and also lost the ability of BAS to be easily combined with other algorithms.

Besides to increase the number of beetles, there are still three directions to improve the searching efficiency of BAS. The first direction is to improve the update method of parameters, such as the step size and the distance between the beetle and the antenna. For example, in the study of Wang and Chen (2018), the step size was updated with a certain possibility only when a better solution was not found. Similarly, Zhao et al. (2020) did not update the step size in their BGA when a better solution was found. The problem of these studies is that many methods and strategies are combined, so it is hard to estimate the effectiveness of the proposed step size update method. In addition, such method, in which the step size is not updated on every iteration, may hinder the convergence process of the algorithm, since the step size may still large at the later stage when the stop criterion is to reach the maximum iteration numbers.

The second direction is to utilize the information about the optimal solutions. For example, in the BGA of Zhao et al. (2020), $(x_{{{\text{gbest}}}}^{{\text{t}}} { - }x^{t} )$ was added into the position update formula of the beetles. By this way, individual beetles would get close to the historical optimal location, thus improving the searching efficiency. However, this method is based on a population of beetles, and it is very likely to fall into local extreme when only a single beetle is utilized.

The last improvement direction is to increase the number of random directions in BAS. For example, Khan et al. (2020) used the adaptive moment estimation (ADAM) (Kingma and Ba 2014) update rule to replace the formula of BAS. Specifically, several random directions were used to generate several solutions. Then, two solution sets contained K minimum or maximum fitness values of the solutions were created and the average of each solution set led to two new vectors. It was believed that a vector calculated by subtraction between the two new vectors represented the gradient of the fitness function at the current position of BAS. Then, the next position was calculated by using ADAM update rule, which was based on the gradient values. However, the validity of the proposed BAS-ADAM algorithm was only verified with several simple models in the study, and the validity of the proposed gradient estimation method was not analyzed. In another study, Zhao et al. (2020) proposed a multi-directional sensing method in their BGA algorithm. Specifically, five directions were generated at each iteration to calculate five next solutions. Then, the best solution among them was chosen as the initial beetle position of the next iteration. However, the validity of the proposed multi-directional sensing method is doubtful, because BGA used a population of beetles and combined many other strategies. Similarly, Wang and Chen (2018) also used multi-directional method in their BSAS algorithm. In BSAS, the number of random directions was increased and an adaptive step size update rule was adopted. It was found that BSAS with more directions (from 1 to 5) resulted in better solutions. However, the biggest problem of these studies is that the rationale of the multi-directional sensing method is not discussed and the criterion to use it is still not clear. For example, we still cannot answer the question: How many directions should be used to search the solution space of a K-dimensional problem.

The purpose of the present study is to improve the usability and the searching efficiency of BAS with three simple modifications. Different from many state-of-the-art algorithms, the most significant feature of the proposed Enhanced Beetle Antenna Search algorithm (EBAS) is that only one beetle is utilized, which is the same as the basic BAS. As a result, the proposed improvement methods ensured that EBAS not only has better searching performance, but also can be easily computed and combined with other algorithms. Specifically, the proposed EBAS contains the following three methods.

First, the step size, which is used to calculate the next solution in BAS, is adaptively calculated according to the needed searching precision and the maximum iteration number. By this way, the parameter uncertainty of BAS is reduced. Furthermore, three step size decreasing methods are compared and the best step size formula is chosen.

Second, a new strategy, which effectively utilizes the information of current iteration, is used to promote the searching ability of BAS. To the best of our knowledge, the left and right solutions calculated from a random direction in each iteration are only used to choose the direction of the next solution in basic BAS and all the existing improved BAS algorithms. The left and right solutions per se are never used to update the next solution or the current optimal solution. However, the next solution calculated by the left or the right solutions is not necessarily superior to the left or the right solutions. When the left or right solutions are better than the next solution or even the current optimal solution, not to use such information will definitely lead to low searching efficiency. The original solution update method of BAS is also not consistent with the foraging behavior of real beetles in nature. If the beetle has found the food by its left or right antenna, it will never jump to the place farther than the position of the antenna. Therefore, in the present contemporary optimal update strategy, the initial solution at each iteration is chosen from the left, the right, and the next solutions of the previous iteration. Furthermore, the current optimal solution is also updated by all the solutions calculated. By this way, the information on each iteration is fully utilized and the performance of BAS is enhanced.

The third and the last improvement of EBAS is the multi-directional sensing method. By calculating multiple pairs of left and right solutions (along with multiple next solutions) on each iteration, EBAS can deal with complex and high-dimensional problems. The multi-directional sensing method is also in line with the nature of the real beetles. Specifically, when a beetle jumps to a location, it can sense the surrounding environment by changing the orientation of its head or antennas. By this way, the beetle can choose the best directions to lead the next movement. In the same way, the beetle in EBAS senses the surrounding solution space by multiple random directions and jumps to the optimal position on each iteration. The present multi-directional sensing method is different from the methods of other studies (Zhao et al. 2020; Wang and Chen 2018) in the following two ways. The first one is that the theoretical rationale and the influential factors of the method are discussed and analyzed in the present study, which is lack in the previous studies. Second, the present multi-directional sensing method can perfectly cooperate with the aforementioned contemporary optimal update strategy. Since the multi-directional sensing method needs to calculate multiple solutions, the contemporary optimal update strategy can fully utilize these solutions and maximize the searching efficiency of the algorithm, such cooperation does not exist in previous studies.

In the last part of the paper, EBAS is compared with many other algorithms to show the superiority of the algorithm in complex and unbiased optimization problems. The unbiased problem means that the solution space of the problem does not contain simple patterns, which may be used to facilitate the searching processes.

## Basic BAS and unbiased test functions

This section will introduce the formula of basic BAS and the utilized test functions.

### Basic BAS algorithm

Assuming the beginning position of the beetle in K-dimensional solution space is $x^{0} = \left( {x_{1} ,\;x_{2} , \ldots ,\;x_{k} } \right)$, set the optimal solution and its fitness as $x^{{\varvec{0}}}$ and $f\left( {x^{0} } \right)$. Then, calculating the left and right solutions by formula (1):

1 $$ \left\{ \begin{array}{lll}{{x_{r} = x^{{{t} }} - d^{t} \cdot \overrightarrow {b} }}\\{{x_{l} = x^{{{t} }} + d^{t} \cdot \overrightarrow {b} }} \end{array} \right. $$

where the subscripts $l$ and $r$ represent the left and right side, respectively, the subscript $t$ represents specific moment during the iteration process. $d^{t}$ represents the distance between the beetle and the antenna at the specific iteration. $\overrightarrow {{\mathbf{b}}}$ is a random direction that represents the direction of the left antenna. The next position of the beetle is calculated by the following formula:

2 $$ x^{t} = x^{t - 1} -\updelta ^{t} \cdot \overrightarrow {b} \cdot sign\left[ {f\left( {x_{l} } \right) - f\left( {x_{r} } \right)} \right] $$

where $f(x)$ is the fitness function of solution $x$, $\updelta ^{t}$ represents the current jump step of the beetle. If $f\left( {x^{t} } \right)$ is better than the fitness of the current optimal solution, the optimal solution and its fitness are updated by $x^{t}$ and $f\left( {x^{t} } \right)$. The formula for $d^{t}$ and $\updelta ^{t}$ are as follows:

3 $$ d^{t} =\upeta _{d} d^{t - 1} + d_{0} \;{\text{or}}\;d^{t} =\updelta ^{t} /c $$

4 $$\updelta ^{t} =\upeta _{\updelta }\updelta ^{t - 1} +\updelta _{0} $$

where $d_{0}$ and $\updelta _{0}$ represent the minimum values of $d^{t}$ and $\updelta ^{t}$, respectively. The initial $\updelta ^{t}$ is usually set as the range of the solution space and the initial $d^{t}$ is usually set as a value less than $\updelta ^{t}$.$\upeta _{d}$ and $\upeta _{\updelta }$ control the decrease rate of $d^{t}$ and $\updelta ^{t}$, a typical value is 0.95. $d^{t}$ can also be set proportional to $\updelta ^{t}$ by a constant parameter $c$, a typical value is 5. $d^{t}$ and $\updelta ^{t}$ are relatively large in the early stage of the optimization process to provide a large searching area, thus increasing the opportunity to find the optimal solution; and the values of $d^{t}$ and $\updelta ^{t}$ are decreased at a later stage to enable the local search and the fast convergence of the algorithm.

### Unbiased test functions

The present study uses the test functions from CEC’17. This test suite and its previous edition (like CEC’15) are designed for single objective real-parameter numerical optimization and have been used by many studies. The name of basic functions in CEC’17 can be seen from Table 1.

**Table 1:** The number and name of basic functions in CEC’17

No	Name	No	Name
$f_{1}$	Bent Cigar	$f_{11}$	High Conditioned Elliptic
$f_{{2}}$	Sum of Different Power	$f_{12}$	Discus
$f_{{3}}$	Zakharov	$f_{13}$	Ackley
$f_{{4}}$	Rosenbrock	$f_{14}$	Weierstrass
$f_{{5}}$	Rastrigin	$f_{15}$	Griewank
$f_{{6}}$	Expanded Schaffer’s F6	$f_{16}$	Katsuura
$f_{7}$	Lunacek bi-Rastrigin	$f_{17}$	HappyCat
$f_{{8}}$	Non-continuous Rotated Rastrigin	$f_{18}$	HGBat
$f_{9}$	Levy	$f_{19}$	Expanded Griewank’s Plus Rosenbrok
$f_{10}$	Modified Schwefel	$f_{20}$	Schaffer’s F7

The above basic functions are shifted, rotated, or mixed to form the actual 30 test functions in CEC’17. The search ranges of the test functions are [-100,100], and the possible dimensions are 2, 10, 30, 50, or 100. In addition, the global optimum of the test functions is related to the function numbers, for example, the optimal solutions of F1 and F3 are 100 and 300, respectively. F1 and F3 are unimodal functions and F4 to F10 are multi-modal functions. F2, which has an unstable behavior, has been removed by the developers of CEC’17 (Awad et al. 2017). F11 to F20 are the hybrid functions which contain different basic functions as subcomponents, thus better simulating the real-world complex optimization problems. F21 to F30 are multi-modal composition functions, in which the hybrid functions are also used as basic functions, therefore, they are even more complex than hybrid functions. The great variety and complexity of the test functions make CEC’17 a best test suite for the present study, since the aim of proposed EBAS is to promote the ability of BAS in solving complex and multi-dimensional problems.

In the literature, many studies directly utilized the basic functions in Table 1 as test functions (e.g., Li et al. 2020; Shao et al. 2018), but the present study used the test functions provided by CEC’17 for the following reasons. First, the test functions in CEC’17 is finely designed and tested, which are more representative and stable. The hybrid and composition functions in it are also much closer to the real-world complex optimization problems. The last and the most important reason is that the test functions in CEC’17 are all shifted, which means its optimal values are not zero and its optimal solutions are not located at the origin of the solution space, and rotated, which means the solution space of the functions is not symmetric. These are very important properties for the test functions to avoid bringing bias into the test. The bias comes from the form of the test functions for many intelligent algorithms. Specifically, when the test function has a global optimal at origin of the solution space or a symmetrical solution space, the convergence speed of many algorithms is much faster than other conditions. This is usually because the design of the formula of the algorithms tends to search the solution space near the origin, or the symmetrical computation process of the algorithms executes quickly when the solution space is also symmetrical. For example, one of the update formulas in SMA (Li et al. 2020) is $\overrightarrow {vc}*\overrightarrow {X}$, while X is the current solution and vc is a random number in [-1, 1], which eventually decreases to zero. The individuals in SMA follow the above update formula are tend to shrink to the origin of the solution space, leading to performance difference according to whether the optimal solution is located at the origin or not. Such bias can be avoided by using the formal test functions of CEC’17.

In the present study, the test functions with 10 and 30 dimensions are used to test the performance of the three improved methods in EBAS, and the test functions with 50 and 100 dimensions are used to compare the effectiveness of EBAS with other competitive algorithms. The best (Fbest), mean (Fmean), and average standard deviation (Std) of the fitness results and the average running time of the algorithms are calculated.

## Adaptive step size reduction method and contemporary optimal update strategy

This section introduces the first and the second improvement methods of EBAS, along with an experimental verification.

### Adaptive step size reduction method

**Fig. 1:**
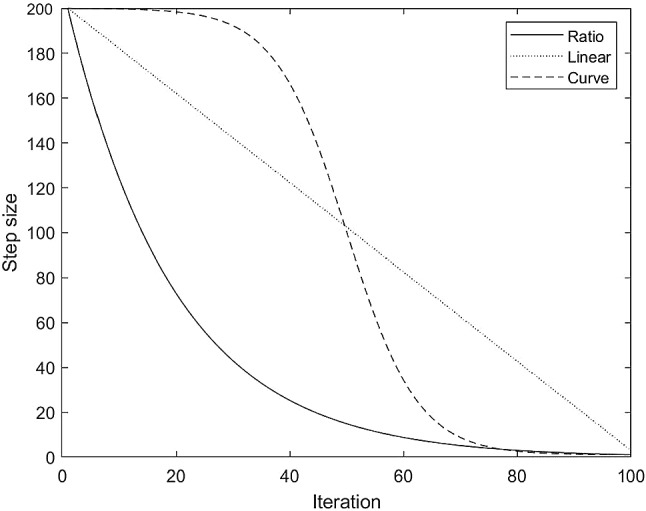
The ratio, linear, and curve methods for step size reduction

The performance of basic BAS is significantly affected by the step size $\updelta ^{t}$ and the decrease rate $\upeta _{\updelta }$. When the decrease rate is large (such as 0.99), the slowly reduced step size is not beneficial for the convergence of the algorithm, and when the decrease rate is small, the rapidly reduced step size is not good for the global optimization process of the algorithm. The existing improved methods related to step size reduction either rely on the overall information of beetle swarm or have uncertainty (Zhao and Qian 2018; Wang and Chen 2018). For example, it is possible that the step size is still large at the later stage of optimization, which is not good for convergence. In EBAS, the step size is controlled according to the maximum value of the optimization range R and the accuracy factor A (e.g., 1/100), so that the step size is orderly reduced from R to the minimum value R * A during the whole iteration process. After determining the maximum and minimum step values, there are also many options to reduce the step size. As shown in Fig. 1 (the solid line), the step size is scaling down in basic BAS, leading to rapid decrement of step size in the early stage. This trend may not be beneficial to the global optimization of the algorithm. So far, there are no researches that have tried other possible step size reduction methods. Therefore, in addition to the basic ratio method, the present study compares two more methods: the linear method and the curve method. As shown in Fig. 1 (the dotted straight line), the linear method means that the step size linearly decreases to the minimum value. In contrast, the dotted curve line in Fig. 1 represents that the step size remains large in the early stage and decreases rapidly in the middle stage, leading to a small step size at the later stage. Theoretically, the curve method can ensure the algorithm to explore a wider solution space in the early stage and accelerate the convergence speed at the later stage, thus balancing the global and local optimization abilities of the algorithm.

The formulas for the three step size reduction methods are as follows:

Ratio:

5 $$ \delta^{t} = \eta_{\delta } *\delta^{t - 1} ,\;\eta_{\delta } = e^{{\left( {{\text{ln}}\left( A \right)/N} \right)}} $$

Linear:

6 $$ \delta^{t} = \delta^{t - 1} - (\delta^{0} - \delta^{0} *A)/N{, } $$

Curve:

7 $$ \delta^{t} = \delta^{0} *A + (\delta^{0} - \delta^{0} *A)/(1 + {\text{e}}^{(8*(2*t/N - 1))} ){,} $$

where $\delta^{t}$ represents the step size at t times iteration, $\delta^{0}$ represents the initial step size, which is set to the maximum optimization range R. In this study, the range of the test functions is [-100, 100], so R is equal to 200. A is the accuracy factor, a typical value is 1/100, which means the step size at the end of the iteration is reduced to 0.01 times of the optimization range R. N is the maximum iteration number.

### Contemporary optimal update strategy

The basic idea of the contemporary optimal update strategy is to select the best solutions at each iteration to update the next position of the beetle and the historical optimal solution. Specifically, the following formula is added after the formulas (1) and (2):

8 $$ x^{*} = \left\{ \begin{gathered} x_{l} {\text{, if min}}\left( {\left[ {f\left( {x_{l} } \right),f\left( {x_{r} } \right),f\left( {x^{t} } \right)} \right]} \right) = f\left( {x_{l} } \right) \hfill \\ x_{r} {\text{, if min}}\left( {\left[ {f\left( {x_{l} } \right),f\left( {x_{r} } \right),f\left( {x^{t} } \right)} \right]} \right) = f\left( {x_{r} } \right) \hfill \\ x^{t} {\text{, if min}}\left( {\left[ {f\left( {x_{l} } \right),f\left( {x_{r} } \right),f\left( {x^{t} } \right)} \right]} \right) = f\left( {x^{t} } \right) \hfill \\ \end{gathered} \right. $$

After the best solution is selected, the historical optimal solution is also updated if the new solution is better. Under this strategy, the number of fitness evaluations remains the same as basic BAS, but the evaluations to the left and right solutions can be fully utilized, thus improving the optimization efficiency without increasing the running time of the algorithm.

### Experimental verification

The effects of the adaptive step size reduction method and the contemporary optimal update strategy are verified by experiments. The test functions are nine functions including F1, F3 to F10 in CEC’17. Algorithms contain basic BAS, and three kinds of step size reduction methods, which are combined or not combined with the contemporary optimal update strategy. As a result, seven algorithms are compared. The parameter settings are as follows: The number of dimensions *K* = 10, maximum iteration number *N* = 100, step size $\delta^{t}$ has an initial value of 200, accuracy factor *A* = 1/100, distance between beetle and antenna $d^{t} = \delta^{t} /{5}$, repetition number of each algorithm is 50, $\upeta _{\updelta }$ is set to 0.95 for basic BAS. To make it fair, the initial random solutions of all algorithms are the same at each repetition. The results can be seen from Table 2.

**Table 2:** The comparison results of basic BAS, three adaptive step size reduction methods with or without the contemporary optimal update strategy

		BAS	Step size method			Optimal update		
			Ratio	Linear	Curve	Ratio	Linear	Curve
F1	Fbest	1.1E + 07	1.9E + 07	4.2E + 08	1.4E + 08	***1.1E* + *06***	*3.9E* + *07*	*3.0E* + *06*
	Fmean	3.3E + 08	4.2E + 08	3.2E + 09	3.9E + 09	***2.5E* + *07***	*1.8E* + *08*	*7.7E* + *07*
	Std	4.2E + 08	5.5E + 08	2.2E + 09	3.1E + 09	***4.5E* + *07***	*1.2E* + *08*	*1.0E* + *08*
	Number of iterations	41.28	44.4	55.68	41.34	85.72	94.94	80.48
F3	Fbest	1.7E + 04	1.8E + 04	***9.4E* + *03***	2.5E + 04	*1.0E* + *04*	1.2E + 04	*2.0E* + *04*
	Fmean	5.5E + 04	5.3E + 04	5.4E + 04	5.6E + 04	*4.7E* + *04*	***4.4E* + *04***	*5.0E* + *04*
	Std	2.6E + 04	2.3E + 04	2.3E + 04	1.8E + 04	*2.1E* + *04*	***1.7E* + *04***	*2.1E* + *04*
	Number of iterations	42.88	44.96	47.98	46.24	82.4	87.5	81.86
F4	Fbest	4.1E + 02	4.1E + 02	4.4E + 02	4.2E + 02	***4.0E* + *02***	*4.1E* + *02*	*4.0E* + *02*
	Fmean	4.8E + 02	4.7E + 02	7.0E + 02	7.1E + 02	***4.4E* + *02***	*4.5E* + *02*	*4.7E* + *02*
	Std	7.0E + 01	5.5E + 01	1.7E + 02	2.6E + 02	***4.2E* + *01***	*4.4E* + *01*	*8.7E* + *01*
	Number of iterations	42.8	46.4	53.76	42.08	83.18	94.5	78.6
F5	Fbest	5.3E + 02	5.5E + 02	5.5E + 02	5.6E + 02	*5.2E* + *02*	*5.3E* + *02*	***5.1E* + *02***
	Fmean	6.2E + 02	6.2E + 02	6.2E + 02	6.2E + 02	*5.6E* + *02*	*5.6E* + *02*	***5.5E* + *02***
	Std	4.1E + 01	3.7E + 01	2.6E + 01	2.6E + 01	*2.4E* + *01*	***1.7E* + *01***	*2.0E* + *01*
	Number of iterations	55.48	57.24	60.82	56.88	90.64	84.46	86.42
F6	Fbest	6.4E + 02	6.4E + 02	6.4E + 02	6.5E + 02	*6.1E* + *02*	*6.1E* + *02*	***6.1E* + *02***
	Fmean	7.0E + 02	7.0E + 02	6.9E + 02	6.9E + 02	*6.5E* + *02*	*6.4E* + *02*	***6.4E* + *02***
	Std	2.8E + 01	2.6E + 01	2.2E + 01	1.6E + 01	*2.5E* + *01*	*1.8E* + *01*	***1.5E* + *01***
	Number of iterations	59.54	59.52	62.08	59.64	87.68	85.46	90.22
F7	Fbest	8.2E + 02	8.0E + 02	8.8E + 02	8.2E + 02	***7.4E* + *02***	*7.6E* + *02*	*7.5E* + *02*
	Fmean	9.8E + 02	9.7E + 02	9.9E + 02	1.1E + 03	*8.1E* + *02*	*8.0E* + *02*	***8.0E* + *02***
	Std	8.0E + 01	6.8E + 01	5.6E + 01	1.1E + 02	*3.4E* + *01*	***1.7E* + *01***	*3.3E* + *01*
	Number of iterations	58.92	59.84	61.38	60.9	88.54	94.28	95.1
F8	Fbest	8.5E + 02	8.5E + 02	8.8E + 02	8.7E + 02	***8.3E* + *02***	*8.4E* + *02*	*8.3E* + *02*
	Fmean	9.1E + 02	9.1E + 02	9.3E + 02	9.2E + 02	*8.6E* + *02*	*8.8E* + *02*	***8.6E* + *02***
	Std	3.3E + 01	3.1E + 01	***1.7E* + *01***	2.5E + 01	*2.4E* + *01*	1.9E + 01	*1.8E* + *01*
	Number of iterations	56.08	58.24	61.46	57.6	91.04	86.82	89.34
F9	Fbest	2.1E + 03	2.6E + 03	2.2E + 03	1.7E + 03	*1.1E* + *03*	*9.9E* + *02*	***9.7E* + *02***
	Fmean	5.8E + 03	5.8E + 03	4.3E + 03	5.3E + 03	*2.6E* + *03*	*2.0E* + *03*	***1.9E* + *03***
	Std	2.3E + 03	2.2E + 03	1.1E + 03	1.6E + 03	*1.0E* + *03*	*8.6E* + *02*	***6.9E* + *02***
	Number of iterations	62.48	63.96	64.28	62.92	78.92	82.4	88.38
F10	Fbest	2.2E + 03	2.2E + 03	2.9E + 03	2.5E + 03	***1.6E* + *03***	*2.2E* + *03*	*2.1E* + *03*
	Fmean	3.0E + 03	3.2E + 03	3.5E + 03	3.2E + 03	***2.5E* + *03***	*3.0E* + *03*	*2.7E* + *03*
	Std	3.8E + 02	*3.9E* + *02*	***2.1E* + *02***	3.6E + 02	4.7E + 02	3.6E + 02	*3.3E* + *02*
	Number of iterations	57.16	59.5	63.4	60.02	89.4	78.3	78.58
Ranking	Fmean	4.5	4.5	6	7	2	3	1

The bold number indicates the best results and the italic number indicates the better results under the same step size reduction methods. Number of iterations means that the left or right solution is better than the next solution in these iterations. The last line indicates the ranking of each algorithm by Friedman test

Several conclusions can be got from the results. First, since the step size reduction trend of the basic BAS is similar to the ratio method (without contemporary optimal update), the searching ability of these two algorithms is almost the same. Second, the algorithms combined with the contemporary optimal update strategy can find better solutions and have lower Std at most of the conditions (indicated by italics), representing the validity of the strategy. Third, as for the three kinds of step size reduction methods, although the performance of the curve method alone is bad, the curve method works well with the contemporary optimal update strategy. Such results may indicate that the basic BAS has strong global searching ability, the rapid reduction in step size by ratio method is needed to turn the global search into local search, thus promoting the convergence speed of the algorithm. The curve method (or the linear method) alone delays the convergence of the algorithm, resulting in bad solutions. However, since the left and right solutions are usually close to the current solution, the global searching ability of the BAS is well controlled by the contemporary optimal update strategy. Consequently, the advantages of the curve (or the linear) method start to contribute to the searching process of the algorithm.

The influence of the contemporary optimal update strategy can also be seen from the number of iterations in Table 2, in which the left or right solution calculated by formula (1) is better than the next solution calculated by formula (2). The number indicates that without the contemporary optimal update strategy, the better left or right solution is discarded at near half of the iterations, no wonder the performance is bad. In contrast, the algorithms with the new strategy have good performance not only because the information of the left and right solutions are utilized, but also because the number of iterations, in which the left or right solution is better, is also increased. This increment of iteration numbers indicates that the new strategy improves the whole searching process, leading the beetle to the global optimal solutions more efficiently.

From the results, we can see that the curve method with the contemporary optimal update strategy has better rank than other conditions, which means the combination of the curve method and the new strategy has better performance than other algorithms. Therefore, the curve method with the new strategy is used in the proposed EBAS algorithm.

Additional analysis indicates that the searching ability of the improved algorithm with curve step size reduction method and contemporary optimal update strategy is not significantly influenced by the value of accuracy factor A. This is different from basic BAS, which is very sensitive to the parameters. The reason for this may be that the curve step size reduction method brings a good balance between global and local search abilities and the contemporary optimal update strategy minimizes the influence of step size by fully utilizing the left and right solutions.

## Multi-directional sensing method

The multi-directional sensing method utilizes multiple random directions, each of them is used to calculate the left, the right, and the next solutions. Integrated with the above contemporary optimal update strategy, the best solutions among them will be selected to be the initial position of the beetle in the next iteration and to update the current optimal solution. By this way, the exploration area is expanded and the possibility to find better solutions around the current beetle position is increased. Similar methods have been used in several previous studies (Zhao et al. 2020; Wang and Chen 2018), however, the present study promotes the usage of the method in several ways. First, the multi-directional sensing method is cooperated with the contemporary optimal update strategy to maximize the profits of the multiple solutions. Second, the rationale of the method is discussed by analyzing two influential factors to provide the instruction about the usage of the method.

When researchers create functional models from specific data, smoothness prior or local constancy prior is the most widely used priori hypothesis. This assumption believes that the results of the functional models should not change greatly in a small area of solution space, which means the small change of the independent variables should not lead to big variations in the results of the test functions. Without this assumption, the functional models cannot represent the trends of the data and predict the possible variation in new data. In the process of BAS optimization, increasing the number of random directions can expand the exploration area of the surrounding space near the current solution. However, under the local constancy priori assumption, the actual effect of the method is affected by many factors.

Two influential factors are considered and analyzed in the present study. The first factor is the similarity between random directions. If the randomly obtained directions are close (in extreme cases, the directions are totally the same or opposite), the fitness values of multiple solutions corresponding to the multiple directions will also be similar. Under such condition, the multi-directional sensing method, which consumes much more calculation time, cannot greatly increase the possibility to find a better solution.

In a solution space, the random directions are represented by vectors. The similarity between two vectors can be represented by the included angle between them. Taking the two-dimensional problems as an example, the angles between two randomly generated vectors are evenly distributed between 0 and PI. When the included angle is PI/2, the two directions are perpendicular (or orthogonal). Then, the two random directions have the greatest difference. The solutions generated by these two directions can cover the largest range of the solution space. However, when the angle between the two vectors is near 0 or PI, the calculated solutions will be very close, leading to similar fitness values according to the local constancy priori hypothesis. Therefore, it is important to make sure the randomly obtained directions have appropriate angles on each other.

The second factor is the problem dimensions. The subsequent analysis indicates that the possibility of two random directions to be orthogonal will increase with the increment of dimensions, which means the contribution of keeping orthogonality to the change of fitness values will decrease with the increment of dimensions. In other words, although the random directions are almost orthogonal for the high-dimensional problems, the variation in the fitness values is limited. If the number of solutions (determined by the number of random directions) in a certain area of the solution space exceeds the number required for the characterization of the fitness values, the effect of the multi-directional sensing method may not further increase with the increment of the random directions. Therefore, theoretically, the effects of the multi-directional sensing method are influenced by two factors: the angles between random directions and the problem dimensions. The actual influence of the two factors will be analyzed in the following part.

### Analysis on the angles of random directions in K-dimensional problems

The angular distribution of two random vectors in K-dimensional space can be derived from the hyper spherical coordinate system, which has the following form:

9 $$ p_{k} (\theta ) = \frac{\Gamma (k/2)}{{\Gamma ((k - 1)/2)\sqrt \pi }}*{\text{sin}}^{k - 2} \theta {,} $$

where $\Gamma$ is the gamma function, $\theta$ is the value of angle. The probability of a specific angle in *K*-dimensional space can be calculated by this formula. However, the formula can only calculate the angular distribution of two random vectors and the calculation process is not intuitive. Therefore, the present study utilizes a simple and intuitive method to calculate the angular distribution among random vectors. By the Monte Carlo method, a large number of random vectors are generated and used to approximately calculate the angular distribution of *M* random vectors in *K*-dimensional space. The dot product formula of two vectors in space is $x^{T} y = \parallel x\parallel_{2} \parallel y\parallel_{2} {\text{cos}}\theta$, it is the product of the L2 norm of the vectors and the included angle. If both vectors are unit vectors, the cosine value of the angle is equal to the dot product of the two vectors with the formula ${\text{cos}}\theta = x^{T} y$. When the two vectors are perpendicular (called orthogonal in multi-dimensional condition), the cosine value of the angle is cos (*PI*/2) = 0, in contrary, the cosine value of the angle is cos (*PI*) = 1 or cos (0) = 1 when the two vectors are opposite or coincided. Therefore, the similarity between two random vectors can be measured by the cosine value of the included angle. In this study, cos (*PI*/4) = 0.7071 (the angle of 45 degrees) to cos (3 * *pi*/4) = -0.7071 (the angle of 135 degrees) is taken as the criterion to indicate that the two directions are not similar. The two directions with cosine values beyond this range are considered to be similar and do not meet the requirements.

**Fig. 2:**
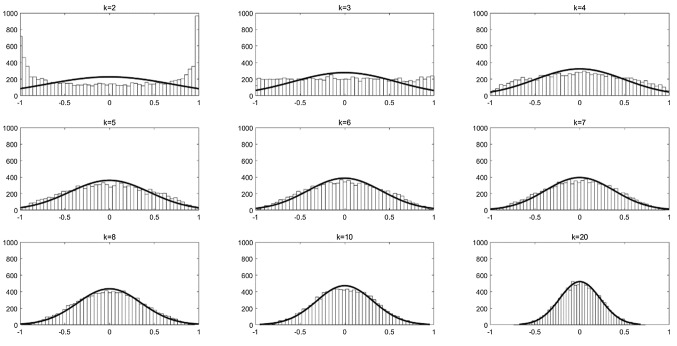
The histogram of the cosine values of the angle between two random unit vectors in K-dimension. The black line indicates the fitting results by the normal distribution. Sampling number is 10000, the bins of the histogram is 50

Figure 2 shows the histogram of the dot product of two random unit vectors (i.e., the cosine values of the angle) after 10,000 samples by Monte Carlo method, while Table 3 shows the ratio of unqualified sampling times to all sampling times under corresponding conditions. Since the angles of two random vectors are evenly distributed in the two-dimensional space, the probability of unqualified sampling will be 50% and this value is almost the same as the ratio value in Table 3, which is 0.5034. In Fig. 2, because the cosine values are a nonlinear function of the angles, the distribution trend of cosine values in two-dimensional space is high at both ends. In the three-dimensional space, the cosine values are evenly distributed, and the proportion of the unqualified sampling is about 29% (see Table 3). In addition, with the increment of dimensions, the cosine values concentrate to the center, and the ratio of unqualified samples decreases continuously, which is less than 5% for the eight-dimensional or higher space. Therefore, under the criterion of 45 to 135 degrees, the two random directions in *K*-dimensional space can be considered to be different when *K* is equal to or greater than eight. The bigger the dimensions are, the more unlikely the two directions are similar. Furthermore, it also can be seen from Fig. 2 that the distribution of cosine values in *K*-dimensional space fits with normal distribution when K is relatively large. Table 3 also includes two fitting parameters based on sampling data: the mean and the standard deviation of the fitted normal distributions. The probabilities of unqualified conditions are also calculated by the probability function of normal distribution with the parameters and the probabilities are very close to the ratio calculated from sampling data, except for the case of *K* = 2 and *K* = 3.

**Table 3:** The ratio of unqualified angles between two random unit vectors in K-dimension and the corresponding fitting parameters of the normal distribution

K	Ratio	Mean	Std	Probability
2	0.5034	−0.0073	0.7109	0.325
3	0.2944	−0.0087	0.5788	0.2276
4	0.185	0.0031	0.4995	0.155
5	0.1248	0.0044	0.4508	0.1144
6	0.081	−0.0038	0.4099	0.0862
7	0.0548	−0.0081	0.3759	0.063
8	0.0348	−0.0047	0.3557	0.0483
10	0.0171	−0.0038	0.3167	0.0264
20	0.0005	−0.0002	0.2221	0.0015

The probability of unqualified angles is calculated by the normal distributions. The number is accurate to the fourth decimal place

When the number of unit vectors is more than two, a new criterion is set. Specifically, the vectors are combined to get all possible pairs of the vectors. If one pair of the vectors is not in the angle range of 45 to 135 degrees, all the vectors are considered as unqualified. In other words, *M* random directions are combined in pairs, if one pair does not meet the requirements, it is considered that these random directions do not meet the requirements. After 10,000 random samplings, Table 4 shows the ratio of unqualified sampling times to all sampling times under different conditions. It can be seen that the higher the dimension is, the more random directions can be used. For example, the space below 10 dimensions only supports two random directions, and the space of 15 dimensions is suitable for six random directions. Therefore, it is not appropriate to use five random directions in five-dimensional and 10-dimensional problems in BGA (Zhao et al. 2020), or to use three, four, or five random directions in nine-dimensional problems in BSAS (Wang and Chen 2018). When the dimension is further increased to 23 or more, even if the same number of random directions as the dimension is used, the proportion of unqualified sampling is lower than 0.05. This result supports the conclusion that almost all random directions in high-dimensional space are orthogonal. Of course, if a narrower criterion (such as 60 to 120 degrees) is used, the qualified dimension number for *M* directions will increase accordingly, but the trend is consistent. That is, a threshold can be found, so that any number of random directions within the dimension range can meet the requirement with a probability greater than 0.95.

**Table 4:** The ratio of unqualified angles among M random directions in K-dimension. Only the ratios near 0.05 are presented

K	M	Ratio	M	Ratio
8	2	0.0398	3	0.103
9	2	0.0233	3	0.0713
10	3	0.0496	4	0.1018
11	3	0.0343	4	0.0649
12	4	0.0500	5	0.0808
13	4	0.0359	5	0.0557
14	5	0.0382	6	0.0529
15	6	0.0416	7	0.0576
16	7	0.0383	8	0.0546
17	9	0.0468	10	0.0607
18	10	0.0441	11	0.0571
19	13	0.0493	14	0.061
20	15	0.0498	16	0.0587
21	17	0.0431	18	0.0522
22	21	0.0462	22	0.0542
23	23	0.0407		
25	25	0.0243		
30	30	0.0058		

### Validation of the multi-directional sensing method and the influence of dimensions

The test functions are nine functions including F1, F3 to F10 in CEC’17. The basic algorithm is the algorithm combined the adaptive curve step size reduction method and the contemporary optimal update strategy.

The number of random directions (*M*) is varied to show the effect of the multi-directional sensing method. The parameter settings are as follows: the number of dimensions *K* = 10 or 30, maximum iteration number *N* = 100, step size $\delta^{t}$ has an initial value of 200, accuracy factor *A *= 1/100, distance between beetle and antenna $d^{t} = \delta^{t} /5$, repetition number of each condition is 50. To make it fair, the initial random solutions of all conditions are the same at each repetition. The results can be seen from Tables 5 and 6. The italicized numbers in the table indicate the optimal value of the results in all cases, while the bold numbers indicate the conditions that further increasing the number of directions does not lead to better results than current ones.

**Table 5:** The results of EBAS with different number of random directions in 10-dimensional problems

M	1	2	3	4	5	6	7	8	9	10	
F1	Fbest	2.2E + 06	1.9E + 05	6.9E + 04	4.4E + 04	**3.0E + 04**	**3.9E + 04**	4.8E + 04	2.8E + 04	***2.7E* + *04***	4.3E + 04
	Fmean	1.5E + 08	3.2E + 06	5.6E + 05	**1.7E + 05**	**1.7E + 05**	2.0E + 05	1.0E + 05	**8.8E + 04**	9.4E + 04	***8.4E* + *04***
	Std	1.6E + 08	3.9E + 06	1.6E + 06	**6.8E + 04**	**2.6E + 05**	5.4E + 05	3.9E + 04	3.0E + 04	2.8E + 04	*2.4E* + *04*
F3	Fbest	2.1E + 04	9.4E + 03	4.3E + 03	4.0E + 03	3.7E + 03	1.8E + 03	**6.0E + 02**	1.0E + 03	***3.0E* + *02***	3.7E + 02
	Fmean	4.6E + 04	3.8E + 04	3.2E + 04	2.6E + 04	1.7E + 04	1.3E + 04	1.0E + 04	1.0E + 04	6.2E + 03	*5.1E* + *03*
	Std	**1.6E + 04**	1.8E + 04	1.3E + 04	1.2E + 04	8.8E + 03	8.4E + 03	**6.8E + 03**	7.7E + 03	6.4E + 03	*5.0E* + *03*
F4	Fbest	4.1E + 02	**4.0E + 02**	4.0E + 02	**4.0E + 02**	4.0E + 02	***4.0E* + *02***	**4.0E + 02**	4.0E + 02	4.0E + 02	4.0E + 02
	Fmean	4.5E + 02	4.3E + 02	4.2E + 02	4.1E + 02	4.1E + 02	***4.1E* + *02***	4.1E + 02	**4.1E + 02**	4.1E + 02	4.1E + 02
	Std	4.6E + 01	3.5E + 01	2.6E + 01	2.2E + 01	1.3E + 01	***1.7E* + *00***	1.4E + 01	**2.6E + 00**	1.5E + 01	1.3E + 01
F5	Fbest	5.1E + 02	**5.1E + 02**	5.1E + 02	**5.1E + 02**	**5.1E + 02**	5.1E + 02	5.1E + 02	***5.1E* + *02***	**5.1E + 02**	5.1E + 02
	Fmean	5.6E + 02	5.3E + 02	5.3E + 02	5.3E + 02	**5.3E + 02**	5.3E + 02	***5.2E* + *02***	**5.2E + 02**	5.2E + 02	5.2E + 02
	Std	2.4E + 01	1.6E + 01	1.5E + 01	1.3E + 01	**1.0E + 01**	1.1E + 01	9.9E + 00	***8.7E* + *00***	**9.8E + 00**	1.2E + 01
F6	Fbest	6.1E + 02	6.0E + 02	**6.0E + 02**	6.0E + 02	6.0E + 02	***6.0E* + *02***	**6.0E + 02**	6.0E + 02	**6.0E + 02**	6.0E + 02
	Fmean	6.4E + 02	6.2E + 02	6.2E + 02	6.1E + 02	6.1E + 02	6.1E + 02	6.1E + 02	***6.1E* + *02***	6.1E + 02	6.1E + 02
	Std	**1.3E + 01**	1.3E + 01	1.1E + 01	**8.5E + 00**	9.5E + 00	8.9E + 00	8.6E + 00	***5.7E* + *00***	7.3E + 00	6.5E + 00
F7	Fbest	7.2E + 02	**7.2E + 02**	7.2E + 02	**7.1E + 02**	7.3E + 02	7.2E + 02	7.1E + 02	***7.1E* + *02***	**7.1E + 02**	7.1E + 02
	Fmean	8.1E + 02	7.6E + 02	7.5E + 02	**7.5E + 02**	7.5E + 02	7.4E + 02	7.4E + 02	7.4E + 02	7.4E + 02	*7.4E* + *02*
	Std	3.4E + 01	2.0E + 01	**1.7E + 01**	1.9E + 01	1.6E + 01	**1.3E + 01**	1.3E + 01	***1.1E* + *01***	**1.3E + 01**	1.4E + 01
F8	Fbest	8.3E + 02	**8.1E + 02**	8.1E + 02	8.1E + 02	**8.1E + 02**	8.1E + 02	8.1E + 02	8.1E + 02	***8.1E* + *02***	8.1E + 02
	Fmean	8.6E + 02	8.4E + 02	8.4E + 02	**8.3E + 02**	8.3E + 02	8.3E + 02	**8.3E + 02**	8.3E + 02	***8.3E* + *02***	8.3E + 02
	Std	**1.8E + 01**	2.0E + 01	1.4E + 01	1.1E + 01	***1.0E* + *01***	1.1E + 01	**1.0E + 01**	1.1E + 01	**1.1E + 01**	1.1E + 01
F9	Fbest	1.0E + 03	9.3E + 02	9.1E + 02	9.0E + 02	9.0E + 02	9.0E + 02	9.0E + 02	**9.0E + 02**	9.0E + 02	*9.0E* + *02*
	Fmean	2.0E + 03	1.3E + 03	1.1E + 03	1.0E + 03	9.8E + 02	9.5E + 02	**9.4E + 02**	9.5E + 02	9.4E + 02	*9.2E* + *02*
	Std	6.5E + 02	2.9E + 02	2.5E + 02	1.7E + 02	1.3E + 02	5.3E + 01	***4.4E* + *01***	**6.3E + 01**	6.4E + 01	4.5E + 01
F10	Fbest	1.8E + 03	**1.4E + 03**	1.5E + 03	1.3E + 03	***1.1E* + *03***	1.4E + 03	**1.3E + 03**	1.5E + 03	**1.4E + 03**	1.5E + 03
	Fmean	2.6E + 03	**2.3E + 03**	2.3E + 03	2.3E + 03	2.1E + 03	2.1E + 03	**2.0E + 03**	2.1E + 03	2.0E + 03	*2.0E* + *03*
	Std	4.3E + 02	3.7E + 02	**3.6E + 02**	**3.7E + 02**	4.4E + 02	3.8E + 02	**3.3E + 02**	3.4E + 02	3.4E + 02	*2.9E* + *02*
Ranking	Fmean	10*	9*	8*	7*	6*	5	3	4	2	1

The last part indicates the ranking of each condition by Friedman test. The ‘*’after the rank indicates significant difference between the algorithm and the rank first algorithm by the post hoc multiple comparisons test

**Table 6:** The results of EBAS with different number of random directions in 30-dimensional problems. The last part indicates the ranking of each condition by Friedman test

M		1	2	3	4	5	10	15	20	25	30
F1	Fbest	1.0E+10	7.7E+08	1.9E+08	8.0E+07	1.8E+07	7.2E+06	1.2E+06	4.1E+05	3.8E+05	*3.0E+05*
	Fmean	2.2E+10	4.7E+09	1.0E+09	3.8E+08	1.6E+08	1.7E+07	8.2E+06	4.6E+06	2.2E+06	*1.1E+06*
	Std	8.4E+09	2.5E+09	6.2E+08	2.4E+08	1.0E+08	8.0E+06	3.7E+06	2.1E+06	1.3E+06	*8.8E+05*
F3	Fbest	**1.4E+05**	**1.4E+05**	1.5E+05	**1.1E+05**	**1.2E+05**	1.3E+05	9.3E+04	**5.7E+04**	7.5E+04	*4.0E+04*
	Fmean	**2.7E+05**	2.7E+05	2.5E+05	2.4E+05	2.3E+05	2.2E+05	1.8E+05	1.6E+05	1.4E+05	*1.1E+05*
	Std	7.0E+04	**6.2E+04**	6.2E+04	5.9E+04	5.7E+04	**4.6E+04**	5.7E+04	4.9E+04	4.0E+04	*3.5E+04*
F4	Fbest	1.2E+03	5.5E+02	5.4E+02	5.3E+02	5.0E+02	**4.8E+02**	**4.8E+02**	4.9E+02	***4.7E+02***	4.7E+02
	Fmean	3.3E+03	1.1E+03	7.3E+02	6.4E+02	6.0E+02	5.5E+02	5.3E+02	5.3E+02	5.2E+02	*5.1E+02*
	Std	1.8E+03	4.5E+02	1.2E+02	6.9E+01	6.1E+01	3.7E+01	3.1E+01	**3.0E+01**	3.3E+01	*2.0E+01*
F5	Fbest	7.2E+02	6.5E+02	**6.3E+02**	6.3E+02	**5.8E+02**	6.1E+02	**5.8E+02**	5.8E+02	5.7E+02	*5.5E+02*
	Fmean	8.8E+02	7.6E+02	7.1E+02	6.9E+02	6.8E+02	6.5E+02	6.4E+02	**6.3E+02**	6.3E+02	*6.1E+02*
	Std	6.9E+01	5.7E+01	**3.9E+01**	4.1E+01	3.6E+01	***3.0E+01***	**3.2E+01**	3.4E+01	3.3E+01	3.2E+01
F6	Fbest	6.6E+02	6.4E+02	6.4E+02	6.4E+02	6.3E+02	6.3E+02	6.2E+02	**6.2E+02**	6.2E+02	*6.2E+02*
	Fmean	7.0E+02	6.8E+02	6.7E+02	6.6E+02	6.6E+02	6.5E+02	6.4E+02	**6.4E+02**	6.4E+02	*6.4E+02*
	Std	1.6E+01	**1.5E+01**	1.5E+01	1.4E+01	1.3E+01	**1.0E+01**	**1.1E+01**	1.1E+01	1.0E+01	*9.7E+00*
F7	Fbest	1.3E+03	9.8E+02	9.4E+02	8.9E+02	8.5E+02	8.4E+02	8.2E+02	***8.0E+02***	8.2E+02	8.1E+02
	Fmean	1.6E+03	1.2E+03	1.1E+03	1.0E+03	9.8E+02	9.2E+02	8.9E+02	8.9E+02	***8.6E+02***	8.7E+02
	Std	2.1E+02	1.1E+02	**6.5E+01**	6.6E+01	5.2E+01	3.8E+01	**3.4E+01**	3.4E+01	*2.6E+01*	3.6E+01
F8	Fbest	9.9E+02	9.4E+02	9.3E+02	9.1E+02	9.0E+02	8.9E+02	***8.6E+02***	8.8E+02	8.7E+02	8.7E+02
	Fmean	1.1E+03	1.1E+03	1.0E+03	1.0E+03	9.8E+02	9.4E+02	9.3E+02	9.3E+02	9.2E+02	*9.2E+02*
	Std	6.9E+01	5.8E+01	**3.7E+01**	4.3E+01	3.7E+01	**3.0E+01**	3.2E+01	2.9E+01	***2.6E+01***	3.0E+01
F9	Fbest	9.8E+03	4.4E+03	4.1E+03	3.6E+03	2.6E+03	**1.7E+03**	2.3E+03	***1.5E+03***	2.0E+03	1.8E+03
	Fmean	2.0E+04	1.2E+04	8.2E+03	7.9E+03	7.3E+03	5.1E+03	4.7E+03	**4.3E+03**	4.6E+03	*3.9E+03*
	Std	6.8E+03	4.1E+03	**2.6E+03**	3.0E+03	2.5E+03	1.6E+03	**1.6E+03**	**1.8E+03**	1.8E+03	*1.4E+03*
F10	Fbest	6.4E+03	5.4E+03	4.4E+03	**4.4E+03**	4.8E+03	**3.6E+03**	3.8E+03	***3.0E+03***	4.1E+03	3.4E+03
	Fmean	7.9E+03	7.0E+03	6.5E+03	6.2E+03	6.1E+03	5.4E+03	5.2E+03	5.1E+03	***5.1E+03***	5.1E+03
	Std	8.5E+02	**7.3E+02**	8.9E+02	**6.5E+02**	**6.7E+02**	7.3E+02	7.0E+02	6.5E+02	***5.7E+02***	6.5E+02
Ranking	Fmean	10*	9*	8*	7*	6*	5*	4*	3	2	1

The ‘*’after the rank indicates significant difference between the algorithm and the rank first algorithm by the post hoc multiple comparisons test

From the results of the 10-dimensional problems, it can be seen that simply increasing the number of random directions from 1 to 2 is enough to get better results. Further increasing the number of random directions (such as 3 or 4) can effectively improve the optimization accuracy and reduce the standard deviation of the results. The running time in the case of *M* = 4 is only about twice that of the case *M* = 1. However, increasing the number of random directions even further does not necessarily improve the optimization results. For example, the results of functions F4 to F8 do not significantly improve after the number of random directions exceeds 4 or 5. Bold values in the table represent the conditions that more directions are used but better solutions (or lower standard deviations) cannot be found. Similarly, the italicized values in the table represent the optimal value of the results in all cases. It can be seen that the optimal values do not always appear at the condition with *M* = 10, though the running time in the case of *M* = 10 is about 3 to 4 times that of the case *M* = 1. This observation is confirmed by the results of Friedman test and post hoc tests. Specifically, the rank first condition is not always the condition with largest M and there is no significant difference between the rank first and the top five conditions according to the results of post hoc multiple comparisons test. In other words, the cost performance will become lower and lower with the increasing of the number of random directions. Referring to the previous analysis of the angular distribution for the 10-dimensional problem, the valid number of random directions is around 3. More random directions do not necessarily bring better results. Along with the experimental results, the analysis suggests 2 to 6 as the best number of random directions in 10-dimensional problems.

Consistent with the 10-dimensional problems, the results of the 30-dimensional problems show that increasing the random directions can effectively improve the optimization accuracy of the algorithm, but this gain effect decreases with the further increment of the random directions. For example, the results of almost all functions in the case of *M* = 2 are better than that in the case of *M* = 1. But for functions F5, F6, and F9, the average fitness in the case of *M* = 20 is better than that in the case of *M* = 25, while for functions F7 and F10, the average fitness in the case of *M* = 25 is better than that in the case of *M* = 30. The running time in the case of *M* = 20 is about seven times that of the case *M* = 1, while the running time in the case of *M* = 30 is about 10 times that of the case *M* = 1. This means that more running time (along with more random directions) do not always bring better results. Although the Friedman test ranks the condition with largest *M* as the best algorithm, the post hoc comparisons do not show significant difference between the rank first and the top three conditions.

Different from the 10-dimensional problems, the analysis results in Table 4 show that even if 30 directions are randomly selected in the 30-dimensional problems, the probability that the directions meet the similarity criterion is only 0.0058. This means the high-dimensional random vectors are almost orthogonal to each other. Therefore, the results confirmed the above discussion about the influence of dimensions on the multi-directional sensing method. Specifically, the variation in the function values is limited in a certain area of solution space. For high-dimensional functions, increasing the number of random directions can indeed increase the chance to find better solution around the current solution, but when the number exceeds the requirements, further increasing the number of directions does not necessarily bring better results. Therefore, it can be concluded that for the 30-dimensional problems, 2 to 20 random directions are a proper number to balance the optimization income and running time of the algorithm. When the optimization result is more important, more random directions can be used; when the running time is more important, the number of random directions can be reduced and even two random directions are enough to bring better results than basic BAS.

### Influence of the combination of the multi-directional sensing method with the other improvement methods

In Sect. 3.3, the influence of the step size reduction methods and the contemporary optimal update strategy is analyzed, the results proved the efficiency of the combination of the curve method and the new strategy. Here, the additional effects of multi-directional sensing method are verified by experiments.

The test functions are nine functions including F1, F3 to F10 in CEC’17. Algorithms contain basic BAS, BAS + multiple sensing, curve method + multiple sensing, strategy + multiple sensing, and condition with all three modifications. The parameter settings are as follows: The number of dimensions *K* = 30, the number of random directions *M* = 10, maximum iteration number *N* = 100, step size $\delta^{t}$ has an initial value of 200, accuracy factor *A* = 1/100, distance between beetle and antenna $d^{t} = \delta^{t} /5$, repetition number of each algorithm is 50, $\upeta _{\updelta }$ is set to 0.95 for basic BAS. To make it fair, the initial random solutions of all algorithms are the same at each repetition. The results can be seen from Table 7.

**Table 7:** The comparison results of the combination of the multi-directional sensing method with the other methods

		BAS	BAS + Multi	Curve + Multi	Strategy + Multi	EBAS
F1	Fbest	1.8E + 10	1.7E + 09	6.2E + 09	**3.3E + 07**	4.7E + 07
	Fmean	3.8E + 10	7.3E + 09	1.4E + 10	2.3E + 08	**1.9E + 08**
	Std	1.2E + 10	3.7E + 09	4.9E + 09	1.9E + 08	**1.4E + 08**
F3	Fbest	1.6E + 05	1.5E + 05	1.5E + 05	1.2E + 05	**1.1E + 05**
	Fmean	2.8E + 05	2.8E + 05	2.7E + 05	2.5E + 05	**2.4E + 05**
	Std	6.3E + 04	7.1E + 04	6.4E + 04	6.5E + 04	**5.7E + 04**
F4	Fbest	2.0E + 03	6.6E + 02	8.5E + 02	5.5E + 02	**5.0E + 02**
	Fmean	7.7E + 03	1.2E + 03	2.0E + 03	6.4E + 02	**6.0E + 02**
	Std	4.5E + 03	4.4E + 02	1.0E + 03	6.8E + 01	**6.2E + 01**
F5	Fbest	8.7E + 02	7.0E + 02	6.8E + 02	6.5E + 02	**5.9E + 02**
	Fmean	1.1E + 03	8.7E + 02	8.3E + 02	7.6E + 02	**6.7E + 02**
	Std	1.3E + 02	8.8E + 01	5.8E + 01	5.3E + 01	**3.7E + 01**
F6	Fbest	7.0E + 02	6.5E + 02	6.5E + 02	6.4E + 02	**6.2E + 02**
	Fmean	7.3E + 02	7.0E + 02	6.9E + 02	6.7E + 02	**6.5E + 02**
	Std	1.9E + 01	1.8E + 01	1.5E + 01	1.7E + 01	**1.3E + 01**
F7	Fbest	2.0E + 03	1.3E + 03	1.3E + 03	9.3E + 02	**8.5E + 02**
	Fmean	2.6E + 03	1.6E + 03	1.7E + 03	1.1E + 03	**9.8E + 02**
	Std	3.1E + 02	1.9E + 02	2.2E + 02	7.7E + 01	**4.8E + 01**
F8	Fbest	1.1E + 03	1.0E + 03	1.0E + 03	9.3E + 02	**9.0E + 02**
	Fmean	1.4E + 03	1.1E + 03	1.1E + 03	1.1E + 03	**9.8E + 02**
	Std	1.1E + 02	7.2E + 01	6.2E + 01	7.3E + 01	**4.0E + 01**
F9	Fbest	2.2E + 04	1.1E + 04	7.3E + 03	**3.8E + 03**	3.8E + 03
	Fmean	4.2E + 04	2.4E + 04	1.9E + 04	1.1E + 04	**6.7E + 03**
	Std	1.1E + 04	7.1E + 03	6.8E + 03	3.9E + 03	**2.3E + 03**
F10	Fbest	7.3E + 03	5.8E + 03	5.5E + 03	**4.2E + 03**	4.3E + 03
	Fmean	9.3E + 03	7.4E + 03	7.2E + 03	**5.8E + 03**	6.0E + 03
	Std	8.1E + 02	8.3E + 02	9.0E + 02	**6.4E + 02**	7.6E + 02
Ranking	Fmean	5	4	3	2	1

The bold number indicates the best results. The last line indicates the ranking of each algorithm by Friedman test

From Table 7, we can see that the accumulation of the three methods gradually increased the searching ability of the algorithm. Specifically, the multi-directional sensing method alone is capable to get better results than basic BAS, the addition of curve step size reduction method or the contemporary optimal update strategy can further promotes the results. The contribution of the new strategy is somehow larger than the curve method, probably because the strategy can maximize the effects of the multiple random directions. In addition, the combination of the three methods results in better performance than any other conditions, thus proving the searching efficiency of the proposed EBAS.

## Pseudo-code of EBAS, complexity analysis, and optimization comparison under the same fitness calculation times

In this section, the pseudo-code and the complexity analysis of the EBAS will be shown first. Then, an additional comparison under the same fitness calculation times is conducted.

### Pseudo-code and complexity of EBAS

The pseudo-code of the EBAS is shown in Algorithm 1.

The time complexity of the basic BAS is *O* (*K* * *N*), *K* is the problem dimension, and *N* is the maximum number of iterations. Among the present three improved methods, only the multi-directional sensing method has an impact on the complexity of the algorithm. After adding this method, the complexity is *O* (*K* * *N* * *M*), and M is the number of random directions. Therefore, when *N* is certain, *M* can be limited, thus controlling the running time of the algorithm.

### Comparison under the same fitness calculation times

Besides reducing the number of random directions, another way to control the running time of the algorithm with the multi-directional sensing method is to increase the number of random directions and reduce the number of iterations simultaneously, thus ensuring the same calculation times of fitness functions. For example, the basic BAS needs to calculate the fitness values three times at each iteration, while the improved algorithm with two random directions needs to calculate six times. If the maximum number of iterations for the improved algorithm is halved, the two algorithms are ensured to have roughly the same calculation number of fitness functions. The optimization results in such case are analyzed and shown in Table 8. The test functions are nine functions including F1, F3 to F10 in CEC’17. The basic algorithm is the algorithm combined the adaptive curve step size reduction method and the contemporary optimal update strategy. The number of random directions M and the iteration number N are varied to ensure the same calculation times of fitness functions. The parameters are set as dimension *K* = 30, the number of random directions M = 1, 2, 4, 5, 8 or 10, the corresponding number of iterations *N* = 1000, 500, 250, 200, 125 or 100, respectively. Therefore, the calculation times of fitness functions for all conditions are all *M* * *N* = 3000. The initial value of step size $\delta^{t}$ is 200, the accuracy factor *A* = 1/100, and distance between beetle and antenna $d^{t} = \delta^{t} /5$. Since the accuracy factor *A* is certain, the optimization accuracy (i.e., the final step size) of the algorithms is not affected by the number of iterations. The repetition number of each algorithm is 50. To make it fair, the initial random solutions of all algorithms are the same at each repetition.

**Table 8:** The results of EBAS with the same calculation times of fitness functions in 30-dimensional problems. The last part indicates the ranking of each condition by Friedman test

		N = 1000, M = 1	N = 500, M = 2	N = 250, M = 4	N = 200, M = 5	N = 125, M = 8	N = 100, M = 10
F1	Fbest	1.4E + 06	7.7E + 05	**5.7E + 05**	8.8E + 05	3.3E + 06	7.1E + 06
	Fmean	2.4E + 06	**1.3E + 06**	2.2E + 06	4.0E + 06	1.1E + 07	1.7E + 07
	Std	6.8E + 05	**3.8E + 05**	1.3E + 06	2.6E + 06	3.6E + 06	6.3E + 06
	Running Time	7.3E-02	4.2E-02	3.2E-02	2.9E-02	2.6E-02	**2.6E−02**
F3	Fbest	9.6E + 04	**8.3E + 04**	1.3E + 05	1.1E + 05	1.2E + 05	1.1E + 05
	Fmean	**1.9E + 05**	2.0E + 05	2.1E + 05	2.2E + 05	2.0E + 05	2.1E + 05
	Std	**3.5E + 04**	4.2E + 04	4.5E + 04	4.8E + 04	4.3E + 04	5.4E + 04
	Running Time	6.6E-02	4.0E-02	3.2E-02	2.8E-02	2.7E-02	**2.5E−02**
F4	Fbest	4.7E + 02	**4.7E + 02**	5.0E + 02	4.9E + 02	4.9E + 02	5.0E + 02
	Fmean	5.2E + 02	**5.2E + 02**	5.3E + 02	5.3E + 02	5.4E + 02	5.5E + 02
	Std	3.2E + 01	3.0E + 01	3.1E + 01	**2.6E + 01**	3.5E + 01	3.8E + 01
	Running Time	6.5E-02	4.0E-02	3.1E-02	2.9E-02	2.6E−02	**2.6E−02**
F5	Fbest	5.9E + 02	6.1E + 02	**5.5E + 02**	5.7E + 02	5.8E + 02	5.8E + 02
	Fmean	7.0E + 02	6.7E + 02	6.5E + 02	6.5E + 02	**6.5E + 02**	6.5E + 02
	Std	4.9E + 01	3.7E + 01	4.5E + 01	3.3E + 01	**3.1E + 01**	3.4E + 01
	Running Time	7.0E-02	4.4E-02	3.4E-02	3.3E-02	2.9E−02	**2.9E−02**
F6	Fbest	6.3E + 02	6.2E + 02	6.2E + 02	6.2E + 02	**6.1E + 02**	6.3E + 02
	Fmean	6.6E + 02	6.4E + 02	**6.4E + 02**	6.4E + 02	6.4E + 02	6.5E + 02
	Std	1.2E + 01	1.1E + 01	**1.0E + 01**	1.1E + 01	1.4E + 01	1.1E + 01
	Running Time	8.3E-02	5.4E-02	4.5E-02	4.4E-02	4.0E-02	**3.9E−02**
F7	Fbest	8.8E + 02	8.8E + 02	8.3E + 02	8.3E + 02	8.4E + 02	**8.1E + 02**
	Fmean	1.1E + 03	9.8E + 02	9.4E + 02	9.2E + 02	9.2E + 02	**9.1E + 02**
	Std	7.3E + 01	6.1E + 01	5.2E + 01	**4.0E + 01**	4.9E + 01	5.3E + 01
	Running Time	7.2E-02	4.4E-02	3.5E-02	3.4E-02	3.0E-02	**2.9E−02**
F8	Fbest	9.2E + 02	**8.7E + 02**	8.8E + 02	8.7E + 02	8.7E + 02	8.8E + 02
	Fmean	1.0E + 03	9.6E + 02	9.4E + 02	9.5E + 02	9.5E + 02	**9.4E + 02**
	Std	4.9E + 01	4.2E + 01	3.9E + 01	3.9E + 01	**3.4E + 01**	3.2E + 01
	Running Time	7.4E-02	4.5E-02	3.5E-02	3.4E-02	3.0E−02	**2.9E−02**
F9	Fbest	4.6E + 03	2.4E + 03	**1.2E + 03**	1.6E + 03	2.2E + 03	2.1E + 03
	Fmean	9.1E + 03	5.7E + 03	**4.3E + 03**	4.3E + 03	4.9E + 03	5.3E + 03
	Std	2.6E + 03	2.3E + 03	1.7E + 03	1.8E + 03	**1.3E + 03**	2.1E + 03
	Running Time	7.1E-02	4.5E-02	3.5E-02	3.3E-02	3.0E−02	**2.9E−02**
F10	Fbest	4.0E + 03	3.9E + 03	3.8E + 03	**3.8E + 03**	4.2E + 03	4.2E + 03
	Fmean	5.7E + 03	5.3E + 03	5.4E + 03	**5.2E + 03**	5.5E + 03	5.5E + 03
	Std	7.7E + 02	6.5E + 02	7.9E + 02	6.5E + 02	6.4E + 02	**6.4E + 02**
	Running Time	7.4E-02	4.6E-02	3.7E-02	3.5E-02	**3.2E−02**	3.2E−02
Ranking	Fmean	6	3	2	1	4	5

It can be seen from the results that, except for function F3, even if the calculation times of fitness functions are the same, increasing the number of random directions can still improve the optimization efficiency and the stability of the algorithm, though this effect tends to lost when the number of iterations is less than 200. In other words, EBAS with more random directions can get better solutions than EBAS with only one random direction by less running time and less iteration number. This result shows that the previous analysis of the effectiveness of the multi-directional sensing method can not only be attributed to the increment of the running time or the more calculation times of the fitness functions. The EBAS does improve the optimization process of the BAS.

## Experimental analysis

Including the above-mentioned experiments, the present study implemented the related algorithms with Matlab2019 and executed the programs in a ThinkPad X1 carbon notebook with i7-8550U CPU and 8G RAM.

### Comparative analysis of EBAS with other algorithms

The comparison algorithms selected in this study are several existing improved BAS algorithms and several representative meta-heuristic algorithms. The existing improved BAS algorithms can be divided into three groups. The first group, which utilizes only one beetle, includes basic BAS (Jiang and Li 2018), QIBAS (Liao and Ouyang 2021), BAS-ADAM (Khan et al. 2020), and BSAS (Wang and Chen 2018); the second group involves a swarm of beetles, including BSO (Wang et al. 2018), LABAS (Xu et al. 2020), and LCCBSA (Zhao and Qian 2018); the third group integrates BAS with other algorithms, such as BGA (Zhao et al. 2020), BFPA (Shao et al. 2018), BGWO (Fan et al. 2021), and SABAS (Zhou et al. 2019). The selected representative meta-heuristic algorithms include several classic algorithms and several state-of-the-art algorithms. The classic algorithms include SA (van Laarhoven and Aarts 1987), GA (Holland 1992), and PSO (Kennedy and Eberhart 1995); the state-of-the-art algorithms include SMA (Li et al. 2020), HHO (Heidari et al. 2019), SSA (Xue and Shen 2020), WOA (Nasiri and Khiyabani 2018), and GWO (Kohli and Arora 2018). Furthermore, in order to show that EBAS can be easily integrated with other algorithms, a new fusion algorithm called EBGWO is also tested. This algorithm is similar to the BGWO, except that EBAS is used to replace the basic BAS in it. By this way, the head wolf of the algorithm gets the ability to obtain prey information from multiple directions through hearing and the contemporary optimal solutions are fully utilized.

The test functions are 29 functions including F1, F3 to F30 in CEC’17. The parameters are set as dimensions *K* = 50 and *K* = 100, the number of iterations *N* = 200, the number of random directions *M* = 30 (for *K* = 50) or 60 (for *K* = 100) for EBAS and EBGWO, step size $\delta^{t}$ has an initial value of 200, accuracy factor A = 1/100, distance between beetle and antenna $d^{t} = \delta^{t} /5$, repetition number of each algorithm is 30. The step size reduction scale factor of basic BAS and other improved BAS algorithms is 0.95, and other parameters (if not listed in Table 9) are the same as EBAS. For the algorithms with a population, the population size is set to 50, the other parameters are shown in Table 9.

**Table 9:** Parameter settings of comparative algorithms

Algorithm	Parameter settings
QIBAS	$\updelta _{0} = 0.01$
BAS_ADAM	${\text{m}} = 10;{\text{k}} = 5;\updelta ^{0} = 10$
BSAS	${\text{m}} = 20;p = 0.8;\updelta _{{^{0} }} = d_{{^{0} }} = eps$
BSO	$V \in [ - 5.12,5.12];\omega \in [0.4,0.9];\lambda = 0.4$
LABAS	$\beta = 1.5;\sigma_{v} = 1;{\text{k}} \in [0,1]$
LCCBAS	$$\begin{gathered} C \in [2.5,1.5];s = 1.5;\mu = 4; \hfill \\ l = 5;\delta = 2.7;m = 30;n = 20 \hfill \\ \end{gathered}$$
BASGA	$$\begin{gathered} n = 10;pe = 0.05;\xi = 10;d^{0} = 0.5; \hfill \\\updelta ^{0} = 5;pc \in [0.6,0.9];pm \in [0.01,0.1] \hfill \\ \end{gathered}$$
BASFPA	$p = 0.8;pl = 0.1;\beta = 1.5;\sigma_{v} = 1$
BGWO	$a \in [0,2];\updelta _{0} = 0.01;r = 2$
SABAS	$T = 200;\alpha = 0.95;L = 100$
SA	$T = 200;\alpha = 0.95$
GA	$pc \in [0.6,0.9];pm \in [0.05,0.2]$
PSO	$c_{1} = 2;c_{2} = 2;vMax = 100$
SMA	$z = 0.03$
HHO	$\beta = 1.5$
SSA	$ST = 0.6;PD = 0.7;SD = 0.2$
WOA	$c_{1} \in [0,1];c_{2} \in [0,1]$
GWO	$a \in [0,2]$
EBGWO	$a \in [0,2]$

As we can see from Tables 10 and 11, EBAS with 30 random directions achieved the best results (including Fbest, Fmean, and Std) among all algorithms in seven functions (F4, F5, F16, F21, F23 to F25). In addition, EBAS got the minimum Fbest values in six functions (F7 to F9, F18, F20, and F29) and got the minimum Fmean values in seven functions (F7 to F9, F10, F22, F27, and F28). Furthermore, the Std result of F28 was also smaller for EBAS than other algorithms. It is worth mentioning that though EBGWO with 30 random directions got better results than many compared algorithms, the total optimization results of it were not superior to EBAS. This is probably because that the framework of EBGWO is still the same as GWO, which may have limited the effects of the additional operations from EBAS.

**Table 10:** The comparative results for 50-dimensional problems in F1-F15

		EBAS	BAS	QIBAS	BAS_ADAM	BSAS	BSO	LABAS	LCCBAS	BASGA	BASFPA	BGWO	SABAS
F1	Fbest	4.8E + 05	9.4E + 10	7.6E + 10	2.4E + 11	2.8E + 09	2.1E + 09	6.1E + 10	1.7E + 09	1.0E + 10	7.5E + 07	1.3E + 10	**1.4E + 02**
	Fmean	6.7E + 05	1.3E + 11	2.2E + 11	3.9E + 11	6.0E + 09	4.4E + 09	8.0E + 10	1.1E + 10	1.7E + 10	4.2E + 08	2.5E + 10	**8.5E + 03**
	Std	1.0E + 05	2.0E + 10	5.4E + 10	9.2E + 10	2.1E + 09	1.5E + 09	9.7E + 09	3.3E + 09	4.5E + 09	2.0E + 08	6.5E + 09	**8.5E + 03**
F3	Fbest	1.5E + 05	3.2E + 05	3.3E + 05	5.3E + 09	1.6E + 05	1.2E + 05	1.1E + 05	1.5E + 05	1.7E + 05	**4.4E + 04**	1.6E + 05	1.3E + 05
	Fmean	2.5E + 05	4.9E + 05	4.7E + 14	1.3E + 16	2.7E + 05	1.6E + 05	1.7E + 05	3.5E + 05	2.4E + 05	**7.0E + 04**	2.3E + 05	1.9E + 05
	Std	6.4E + 04	1.0E + 05	2.1E + 15	2.7E + 16	6.0E + 04	3.1E + 04	2.6E + 04	3.3E + 05	5.5E + 04	**1.6E + 04**	3.8E + 04	4.0E + 04
F4	Fbest	**4.9E + 02**	9.8E + 03	3.4E + 04	6.5E + 04	8.3E + 02	8.5E + 02	1.3E + 04	9.4E + 02	1.6E + 03	6.6E + 02	2.6E + 03	5.9E + 02
	Fmean	**6.1E + 02**	3.3E + 04	8.3E + 04	2.7E + 05	1.1E + 03	1.2E + 03	2.0E + 04	1.9E + 03	3.0E + 03	7.9E + 02	5.0E + 03	1.2E + 03
	Std	**5.6E + 01**	1.2E + 04	3.9E + 04	1.1E + 05	2.6E + 02	1.8E + 02	3.4E + 03	5.6E + 02	7.3E + 02	7.9E + 01	1.3E + 03	4.3E + 02
F5	Fbest	**6.4E + 02**	1.4E + 03	1.3E + 03	1.6E + 03	9.8E + 02	7.5E + 02	1.1E + 03	8.3E + 02	7.4E + 02	7.3E + 02	8.3E + 02	1.1E + 03
	Fmean	**7.3E + 02**	1.7E + 03	1.6E + 03	2.1E + 03	1.1E + 03	8.5E + 02	1.1E + 03	9.6E + 02	8.2E + 02	8.7E + 02	9.4E + 02	1.3E + 03
	Std	**4.2E + 01**	1.6E + 02	1.8E + 02	2.5E + 02	7.7E + 01	5.2E + 01	3.3E + 01	7.6E + 01	4.2E + 01	6.5E + 01	3.9E + 01	1.3E + 02
F6	Fbest	6.3E + 02	7.0E + 02	6.9E + 02	7.2E + 02	6.5E + 02	6.3E + 02	6.7E + 02	6.6E + 02	6.4E + 02	6.4E + 02	6.6E + 02	6.9E + 02
	Fmean	6.5E + 02	7.4E + 02	7.2E + 02	7.8E + 02	6.9E + 02	6.5E + 02	6.9E + 02	6.7E + 02	6.5E + 02	6.6E + 02	6.7E + 02	7.1E + 02
	Std	1.0E + 01	1.5E + 01	1.5E + 01	2.2E + 01	2.3E + 01	1.2E + 01	5.8E + 00	6.5E + 00	5.3E + 00	9.8E + 00	6.7E + 00	1.6E + 01
F7	Fbest	**9.2E + 02**	3.5E + 03	4.7E + 03	5.9E + 03	1.4E + 03	1.1E + 03	1.8E + 03	1.8E + 03	1.3E + 03	1.1E + 03	1.5E + 03	1.6E + 03
	Fmean	**1.0E + 03**	4.9E + 03	6.4E + 03	8.3E + 03	1.5E + 03	1.3E + 03	2.0E + 03	2.6E + 03	1.5E + 03	1.3E + 03	1.7E + 03	2.3E + 03
	Std	6.4E + 01	7.7E + 02	8.1E + 02	1.1E + 03	9.1E + 01	8.0E + 01	1.3E + 02	3.3E + 02	1.3E + 02	1.3E + 02	1.0E + 02	4.7E + 02
F8	Fbest	**9.3E + 02**	1.7E + 03	1.5E + 03	2.1E + 03	1.2E + 03	1.1E + 03	1.3E + 03	1.2E + 03	1.0E + 03	1.1E + 03	1.2E + 03	1.4E + 03
	Fmean	**1.0E + 03**	2.0E + 03	1.9E + 03	2.4E + 03	1.4E + 03	1.2E + 03	1.4E + 03	1.3E + 03	1.1E + 03	1.2E + 03	1.2E + 03	1.7E + 03
	Std	4.7E + 01	1.4E + 02	2.2E + 02	1.9E + 02	6.2E + 01	5.0E + 01	3.7E + 01	6.8E + 01	4.8E + 01	7.1E + 01	4.5E + 01	1.3E + 02
F9	Fbest	**3.4E + 03**	4.4E + 04	2.6E + 04	6.6E + 04	1.9E + 04	4.9E + 03	2.3E + 04	1.5E + 04	6.8E + 03	9.3E + 03	1.4E + 04	2.2E + 04
	Fmean	**7.7E + 03**	6.9E + 04	5.4E + 04	1.1E + 05	5.0E + 04	1.2E + 04	3.0E + 04	2.1E + 04	1.2E + 04	1.4E + 04	1.8E + 04	3.0E + 04
	Std	2.6E + 03	1.6E + 04	2.7E + 04	2.3E + 04	2.3E + 04	3.7E + 03	4.5E + 03	4.5E + 03	2.3E + 03	3.9E + 03	2.6E + 03	5.4E + 03
F10	Fbest	6.1E + 03	1.3E + 04	9.4E + 03	1.7E + 04	1.3E + 04	1.2E + 04	1.4E + 04	7.1E + 03	6.6E + 03	6.3E + 03	8.3E + 03	7.2E + 03
	Fmean	**7.4E + 03**	1.4E + 04	1.3E + 04	1.9E + 04	1.5E + 04	1.4E + 04	1.5E + 04	9.9E + 03	8.2E + 03	9.3E + 03	1.0E + 04	9.8E + 03
	Std	7.6E + 02	9.4E + 02	1.7E + 03	9.6E + 02	8.8E + 02	1.1E + 03	4.1E + 02	2.4E + 03	7.7E + 02	1.1E + 03	9.0E + 02	1.4E + 03
F11	Fbest	1.4E + 03	3.8E + 04	3.0E + 04	1.7E + 06	2.5E + 03	3.1E + 03	1.5E + 04	1.8E + 03	7.3E + 03	1.6E + 03	9.7E + 03	1.4E + 03
	Fmean	1.7E + 03	8.9E + 04	1.3E + 09	2.2E + 11	3.6E + 03	9.0E + 03	2.0E + 04	2.4E + 03	1.9E + 04	2.0E + 03	1.7E + 04	1.7E + 03
	Std	1.5E + 02	2.8E + 04	5.4E + 09	4.2E + 11	8.8E + 02	4.4E + 03	3.4E + 03	4.1E + 02	8.5E + 03	2.0E + 02	3.2E + 03	2.3E + 02
F12	Fbest	2.4E + 07	1.4E + 10	1.6E + 10	1.2E + 11	4.5E + 08	2.7E + 08	1.9E + 10	1.1E + 08	2.8E + 08	5.4E + 07	9.0E + 08	**1.4E + 06**
	Fmean	7.9E + 07	3.0E + 10	1.0E + 11	2.7E + 11	1.2E + 09	6.8E + 08	3.7E + 10	7.5E + 08	1.2E + 09	2.8E + 08	3.1E + 09	**1.7E + 07**
	Std	3.8E + 07	1.1E + 10	6.4E + 10	6.8E + 10	3.7E + 08	3.0E + 08	7.3E + 09	8.7E + 08	7.8E + 08	2.1E + 08	1.7E + 09	**1.2E + 07**
F13	Fbest	7.6E + 04	2.2E + 07	1.6E + 06	1.2E + 11	1.3E + 08	2.8E + 05	7.5E + 09	3.7E + 05	3.7E + 05	**4.3E + 04**	3.4E + 05	5.7E + 04
	Fmean	2.4E + 05	6.1E + 09	3.5E + 10	2.5E + 11	3.6E + 08	5.2E + 06	1.7E + 10	6.9E + 05	2.1E + 07	**1.7E + 05**	6.6E + 07	2.2E + 05
	Std	1.2E + 05	5.8E + 09	3.8E + 10	7.8E + 10	2.0E + 08	5.4E + 06	5.6E + 09	1.7E + 05	4.2E + 07	1.3E + 05	8.1E + 07	1.8E + 05
F14	Fbest	2.7E + 04	2.9E + 06	2.6E + 06	4.7E + 08	3.2E + 05	3.4E + 04	3.1E + 06	2.5E + 04	1.7E + 05	2.2E + 04	3.1E + 05	**1.0E + 04**
	Fmean	1.6E + 05	6.8E + 07	3.8E + 08	4.2E + 09	9.9E + 05	5.4E + 05	2.6E + 07	1.3E + 07	3.5E + 06	1.4E + 05	6.8E + 06	**9.9E + 04**
	Std	1.0E + 05	7.0E + 07	5.5E + 08	3.2E + 09	4.8E + 05	5.7E + 05	2.3E + 07	4.6E + 07	2.6E + 06	9.8E + 04	6.5E + 06	**6.4E + 04**
F15	Fbest	4.0E + 04	5.5E + 04	2.8E + 04	3.6E + 10	3.5E + 07	**9.2E + 03**	4.6E + 08	1.4E + 05	2.3E + 04	1.4E + 04	1.7E + 04	3.1E + 04
	Fmean	1.1E + 05	8.2E + 08	2.8E + 09	1.0E + 11	1.3E + 08	6.3E + 04	2.1E + 09	2.1E + 05	1.8E + 05	5.6E + 04	1.8E + 05	1.0E + 05
	Std	8.4E + 04	1.9E + 09	7.5E + 09	3.6E + 10	7.6E + 07	3.9E + 04	1.4E + 09	5.2E + 04	3.1E + 05	3.9E + 04	3.1E + 05	5.9E + 04

		SA	GA	PSO	SMA	HHO	SSA	WOA	GWO	EBGWO
F1	best	1.5E + 11	4.5E + 10	1.8E + 09	2.5E + 07	1.2E + 10	6.2E + 10	2.0E + 10	6.1E + 10	3.4E + 05
	Mean	1.7E + 11	6.3E + 10	3.2E + 09	4.7E + 07	2.8E + 10	7.9E + 10	3.3E + 10	9.6E + 10	5.9E + 05
	Std	8.8E + 09	7.7E + 09	1.2E + 09	1.4E + 07	6.5E + 09	8.8E + 09	7.0E + 09	1.2E + 10	9.7E + 04
F3	best	2.4E + 05	2.1E + 05	1.9E + 05	1.6E + 05	1.5E + 05	2.0E + 05	1.9E + 05	1.9E + 05	6.4E + 04
	Mean	3.0E + 05	3.2E + 05	2.9E + 05	2.9E + 05	1.9E + 05	3.2E + 05	3.4E + 05	2.5E + 05	9.3E + 04
	Std	3.7E + 04	7.8E + 04	5.7E + 04	7.8E + 04	2.2E + 04	7.5E + 04	1.1E + 05	4.1E + 04	2.2E + 04
F4	best	3.9E + 04	8.9E + 03	8.9E + 02	5.5E + 02	2.3E + 03	1.3E + 04	4.3E + 03	1.4E + 04	5.4E + 02
	Mean	4.9E + 04	1.3E + 04	1.1E + 03	6.7E + 02	5.8E + 03	2.1E + 04	7.2E + 03	2.7E + 04	6.6E + 02
	Std	5.3E + 03	2.3E + 03	1.6E + 02	6.2E + 01	1.6E + 03	3.7E + 03	1.8E + 03	7.3E + 03	7.6E + 01
F5	best	1.4E + 03	1.1E + 03	8.7E + 02	7.0E + 02	9.2E + 02	9.1E + 02	1.0E + 03	1.1E + 03	7.1E + 02
	Mean	1.4E + 03	1.1E + 03	9.4E + 02	8.1E + 02	9.7E + 02	1.1E + 03	1.1E + 03	1.1E + 03	8.0E + 02
	Std	3.7E + 01	2.7E + 01	3.7E + 01	5.1E + 01	3.3E + 01	6.0E + 01	7.7E + 01	4.4E + 01	4.7E + 01
F6	best	7.1E + 02	6.9E + 02	**6.2E + 02**	6.2E + 02	6.7E + 02	6.7E + 02	6.8E + 02	6.7E + 02	6.3E + 02
	Mean	7.2E + 02	7.0E + 02	**6.3E + 02**	6.5E + 02	6.8E + 02	6.9E + 02	7.0E + 02	6.9E + 02	6.5E + 02
	Std	**4.2E + 00**	4.8E + 00	5.9E + 00	1.3E + 01	6.8E + 00	7.2E + 00	1.2E + 01	7.1E + 00	8.3E + 00
F7	best	3.9E + 03	1.6E + 03	1.3E + 03	1.0E + 03	1.7E + 03	1.9E + 03	1.7E + 03	1.8E + 03	1.0E + 03
	Mean	4.6E + 03	1.8E + 03	1.4E + 03	1.2E + 03	1.9E + 03	1.9E + 03	1.9E + 03	2.0E + 03	1.1E + 03
	Std	2.2E + 02	8.4E + 01	6.7E + 01	8.5E + 01	8.8E + 01	**3.1E + 01**	1.1E + 02	6.8E + 01	1.2E + 02
F8	best	1.6E + 03	1.4E + 03	1.1E + 03	1.0E + 03	1.2E + 03	1.3E + 03	1.3E + 03	1.3E + 03	1.0E + 03
	Mean	1.7E + 03	1.4E + 03	1.2E + 03	1.1E + 03	1.3E + 03	1.4E + 03	1.5E + 03	1.4E + 03	1.1E + 03
	Std	4.0E + 01	**2.8E + 01**	4.0E + 01	5.4E + 01	4.4E + 01	5.9E + 01	1.0E + 02	4.9E + 01	4.1E + 01
F9	best	5.1E + 04	1.9E + 04	3.9E + 03	9.7E + 03	2.6E + 04	1.9E + 04	2.2E + 04	2.7E + 04	7.7E + 03
	Mean	6.3E + 04	2.6E + 04	7.9E + 03	1.9E + 04	3.2E + 04	2.3E + 04	3.8E + 04	3.5E + 04	1.2E + 04
	Std	4.8E + 03	4.3E + 03	4.0E + 03	4.4E + 03	3.3E + 03	3.0E + 03	9.4E + 03	4.3E + 03	**2.0E + 03**
F10	best	1.4E + 04	1.4E + 04	1.3E + 04	**5.1E + 03**	8.7E + 03	1.1E + 04	1.2E + 04	1.3E + 04	6.5E + 03
	Mean	1.6E + 04	1.5E + 04	1.5E + 04	8.1E + 03	1.1E + 04	1.3E + 04	1.4E + 04	1.5E + 04	7.7E + 03
	Std	**3.7E + 02**	6.7E + 02	7.9E + 02	1.1E + 03	1.0E + 03	8.8E + 02	1.2E + 03	7.4E + 02	8.8E + 02
F11	best	2.5E + 04	1.1E + 04	2.7E + 03	**1.4E + 03**	3.6E + 03	1.1E + 04	6.7E + 03	2.1E + 04	1.4E + 03
	Mean	3.5E + 04	2.1E + 04	4.5E + 03	1.6E + 03	6.5E + 03	1.9E + 04	1.1E + 04	2.7E + 04	**1.5E + 03**
	Std	5.4E + 03	6.5E + 03	1.5E + 03	1.7E + 02	1.6E + 03	3.5E + 03	2.2E + 03	2.9E + 03	**8.3E + 01**
F12	best	4.2E + 10	1.5E + 10	7.3E + 07	1.2E + 07	1.9E + 09	2.2E + 10	2.1E + 09	2.6E + 10	2.4E + 07
	Mean	6.5E + 10	2.4E + 10	2.7E + 08	5.6E + 07	8.6E + 09	4.3E + 10	7.5E + 09	4.9E + 10	1.1E + 08
	Std	7.4E + 09	4.5E + 09	1.6E + 08	2.5E + 07	3.5E + 09	9.3E + 09	2.3E + 09	1.3E + 10	4.8E + 07
F13	best	1.3E + 10	1.9E + 09	2.2E + 05	6.5E + 04	4.1E + 07	2.8E + 09	2.2E + 08	5.6E + 09	7.8E + 04
	Mean	2.5E + 10	4.9E + 09	2.9E + 06	2.3E + 05	6.9E + 08	1.2E + 10	1.0E + 09	2.0E + 10	1.9E + 05
	Std	5.1E + 09	2.1E + 09	8.7E + 06	1.5E + 05	6.3E + 08	6.9E + 09	6.3E + 08	9.2E + 09	**9.3E + 04**
F14	best	7.0E + 06	1.5E + 06	2.1E + 05	9.2E + 04	1.6E + 06	5.7E + 05	1.1E + 06	2.0E + 06	4.2E + 04
	Mean	3.1E + 07	1.7E + 07	1.4E + 06	1.4E + 06	1.1E + 07	2.5E + 07	8.9E + 06	4.7E + 07	3.4E + 05
	Std	1.3E + 07	1.2E + 07	1.2E + 06	1.1E + 06	9.7E + 06	2.5E + 07	8.9E + 06	5.2E + 07	2.9E + 05
F15	best	4.4E + 09	1.8E + 08	1.3E + 04	1.4E + 04	1.1E + 06	2.2E + 08	1.2E + 07	4.5E + 08	2.5E + 04
	Mean	7.5E + 09	6.1E + 08	7.4E + 05	**4.6E + 04**	5.3E + 07	1.6E + 09	1.3E + 08	3.5E + 09	6.8E + 04
	Std	1.7E + 09	5.4E + 08	2.7E + 06	**1.8E + 04**	1.4E + 08	1.4E + 09	9.2E + 07	2.2E + 09	3.8E + 04

**Table 11:** The comparative results for 50-dimensional problems in F16-F30

		EBAS	BAS	QIBAS	BAS_ADAM	BSAS	BSO	LABAS	LCCBAS	BASGA	BASFPA	BGWO	SABAS
F16	Fbest	**2.6E + 03**	6.0E + 03	7.0E + 03	1.9E + 04	3.9E + 03	2.9E + 03	5.5E + 03	3.6E + 03	3.2E + 03	3.2E + 03	3.9E + 03	3.3E + 03
	Fmean	**3.3E + 03**	8.0E + 03	1.6E + 04	5.6E + 04	5.2E + 03	3.7E + 03	7.1E + 03	4.7E + 03	4.1E + 03	4.2E + 03	5.2E + 03	5.2E + 03
	Std	**4.2E + 02**	1.5E + 03	8.1E + 03	3.3E + 04	5.4E + 02	4.7E + 02	6.1E + 02	5.6E + 02	4.6E + 02	5.9E + 02	5.9E + 02	8.9E + 02
F17	Fbest	2.5E + 03	4.4E + 03	4.3E + 03	3.7E + 06	3.9E + 03	2.7E + 03	3.9E + 03	3.2E + 03	3.1E + 03	2.5E + 03	2.9E + 03	3.2E + 03
	Fmean	3.2E + 03	6.8E + 03	1.8E + 04	3.6E + 08	4.6E + 03	3.3E + 03	5.0E + 03	4.0E + 03	3.7E + 03	3.7E + 03	4.0E + 03	4.1E + 03
	Std	3.5E + 02	3.3E + 03	3.8E + 04	5.7E + 08	3.6E + 02	**2.7E + 02**	5.8E + 02	4.3E + 02	3.8E + 02	4.9E + 02	4.3E + 02	5.8E + 02
F18	Fbest	**1.6E + 05**	3.8E + 06	1.8E + 07	9.0E + 08	2.2E + 06	2.3E + 05	1.1E + 07	2.6E + 05	2.0E + 06	1.8E + 05	4.1E + 06	2.0E + 05
	Fmean	2.3E + 06	1.2E + 08	1.2E + 09	2.2E + 10	7.7E + 06	2.7E + 06	5.8E + 07	2.2E + 07	2.3E + 07	1.5E + 06	5.3E + 07	**1.2E + 06**
	Std	2.3E + 06	8.2E + 07	2.1E + 09	1.6E + 10	3.9E + 06	2.3E + 06	2.5E + 07	8.7E + 07	1.4E + 07	1.1E + 06	3.8E + 07	**9.3E + 05**
F19	Fbest	3.1E + 05	5.9E + 05	5.8E + 06	2.4E + 10	3.4E + 07	5.4E + 04	2.6E + 08	1.0E + 05	3.1E + 04	1.9E + 05	3.7E + 04	5.3E + 05
	Fmean	4.4E + 06	2.3E + 08	2.8E + 09	4.2E + 10	7.2E + 07	2.8E + 06	9.3E + 08	4.1E + 06	1.0E + 06	4.8E + 06	5.0E + 06	1.3E + 06
	Std	3.3E + 06	3.5E + 08	8.5E + 09	1.4E + 10	3.5E + 07	3.0E + 06	4.3E + 08	3.2E + 06	1.4E + 06	3.2E + 06	7.5E + 06	3.4E + 05
F20	Fbest	**2.5E + 03**	3.6E + 03	3.1E + 03	5.0E + 03	3.6E + 03	2.8E + 03	3.7E + 03	3.4E + 03	2.7E + 03	2.9E + 03	2.9E + 03	3.2E + 03
	Fmean	3.1E + 03	4.5E + 03	4.4E + 03	6.1E + 03	4.3E + 03	3.4E + 03	4.1E + 03	4.0E + 03	3.3E + 03	3.5E + 03	3.5E + 03	4.2E + 03
	Std	3.3E + 02	3.9E + 02	5.8E + 02	4.6E + 02	4.1E + 02	4.2E + 02	2.0E + 02	5.2E + 02	3.2E + 02	3.6E + 02	3.2E + 02	4.7E + 02
F21	Fbest	**2.5E + 03**	3.0E + 03	3.0E + 03	3.5E + 03	2.8E + 03	2.6E + 03	2.9E + 03	2.7E + 03	2.5E + 03	2.6E + 03	2.7E + 03	2.8E + 03
	Fmean	**2.5E + 03**	3.4E + 03	3.6E + 03	4.5E + 03	2.9E + 03	2.6E + 03	3.0E + 03	2.8E + 03	2.6E + 03	2.7E + 03	2.8E + 03	3.1E + 03
	Std	**3.6E + 01**	1.8E + 02	4.3E + 02	1.4E + 03	5.4E + 01	4.2E + 01	4.6E + 01	8.8E + 01	5.2E + 01	6.5E + 01	5.9E + 01	1.5E + 02
F22	Fbest	8.1E + 03	1.4E + 04	1.0E + 04	1.9E + 04	1.5E + 04	1.4E + 04	1.6E + 04	9.1E + 03	8.7E + 03	9.1E + 03	1.0E + 04	8.5E + 03
	Fmean	**9.2E + 03**	1.6E + 04	1.6E + 04	2.1E + 04	1.6E + 04	1.6E + 04	1.7E + 04	1.1E + 04	1.1E + 04	1.1E + 04	1.3E + 04	1.1E + 04
	Std	6.3E + 02	1.2E + 03	2.4E + 03	1.0E + 03	8.1E + 02	1.0E + 03	4.0E + 02	1.7E + 03	9.8E + 02	1.0E + 03	1.1E + 03	1.7E + 03
F23	Fbest	**2.9E + 03**	3.8E + 03	4.0E + 03	4.9E + 03	3.2E + 03	3.1E + 03	3.8E + 03	3.4E + 03	3.1E + 03	3.1E + 03	3.3E + 03	3.4E + 03
	Fmean	**3.0E + 03**	5.0E + 03	5.6E + 03	7.5E + 03	3.3E + 03	3.3E + 03	4.1E + 03	4.1E + 03	3.3E + 03	3.3E + 03	3.6E + 03	3.8E + 03
	Std	**4.5E + 01**	7.6E + 02	8.0E + 02	1.7E + 03	6.0E + 01	1.2E + 02	1.5E + 02	2.8E + 02	1.0E + 02	1.0E + 02	1.5E + 02	2.8E + 02
F24	Fbest	**3.0E + 03**	4.0E + 03	4.8E + 03	4.9E + 03	3.3E + 03	3.4E + 03	4.2E + 03	4.0E + 03	3.3E + 03	3.2E + 03	3.4E + 03	3.3E + 03
	Fmean	**3.1E + 03**	4.6E + 03	6.2E + 03	8.4E + 03	3.4E + 03	3.7E + 03	4.6E + 03	4.5E + 03	3.5E + 03	3.5E + 03	3.8E + 03	3.7E + 03
	Std	**4.4E + 01**	4.5E + 02	7.7E + 02	1.8E + 03	7.2E + 01	1.9E + 02	2.2E + 02	3.6E + 02	8.3E + 01	1.3E + 02	1.9E + 02	2.2E + 02
F25	Fbest	**3.0E + 03**	1.4E + 04	1.2E + 04	6.3E + 04	3.3E + 03	3.3E + 03	8.1E + 03	3.4E + 03	4.0E + 03	3.2E + 03	4.6E + 03	3.1E + 03
	Fmean	**3.1E + 03**	2.2E + 04	4.0E + 04	1.4E + 05	3.5E + 03	3.6E + 03	1.2E + 04	3.9E + 03	4.9E + 03	3.3E + 03	5.9E + 03	3.7E + 03
	Std	**3.1E + 01**	6.3E + 03	1.9E + 04	6.4E + 04	1.7E + 02	1.9E + 02	1.5E + 03	5.0E + 02	6.9E + 02	1.1E + 02	8.1E + 02	6.0E + 02
F26	Fbest	3.5E + 03	1.4E + 04	1.9E + 04	2.6E + 04	4.1E + 03	5.8E + 03	1.4E + 04	1.3E + 04	9.7E + 03	4.5E + 03	1.1E + 04	**2.9E + 03**
	Fmean	6.3E + 03	2.1E + 04	3.8E + 04	6.6E + 04	9.3E + 03	8.7E + 03	1.6E + 04	1.7E + 04	1.2E + 04	9.1E + 03	1.3E + 04	1.4E + 04
	Std	7.0E + 02	3.9E + 03	1.4E + 04	2.8E + 04	1.1E + 03	1.3E + 03	1.2E + 03	2.0E + 03	1.0E + 03	1.5E + 03	1.1E + 03	3.2E + 03
F27	Fbest	3.3E + 03	4.9E + 03	**3.2E + 03**	1.1E + 04	3.5E + 03	3.6E + 03	5.9E + 03	4.0E + 03	3.9E + 03	3.5E + 03	3.9E + 03	3.6E + 03
	Fmean	**3.5E + 03**	6.1E + 03	8.9E + 03	2.1E + 04	3.6E + 03	4.3E + 03	6.6E + 03	5.6E + 03	4.4E + 03	3.9E + 03	4.6E + 03	4.4E + 03
	Std	9.7E + 01	1.0E + 03	6.1E + 03	7.0E + 03	**9.0E + 01**	5.1E + 02	5.2E + 02	1.0E + 03	2.4E + 02	2.3E + 02	4.3E + 02	6.7E + 02
F28	Fbest	3.3E + 03	9.1E + 03	3.3E + 03	3.0E + 04	3.5E + 03	3.8E + 03	7.7E + 03	4.2E + 03	4.6E + 03	3.5E + 03	5.5E + 03	**3.3E + 03**
	Fmean	**3.4E + 03**	1.4E + 04	9.6E + 03	5.2E + 04	3.7E + 03	6.5E + 03	1.0E + 04	6.9E + 03	5.6E + 03	3.9E + 03	6.6E + 03	4.2E + 03
	Std	**3.2E + 01**	2.7E + 03	1.4E + 04	1.9E + 04	1.2E + 02	4.0E + 03	1.1E + 03	4.8E + 03	4.9E + 02	2.7E + 02	6.0E + 02	1.1E + 03
F29	Fbest	**4.4E + 03**	8.6E + 03	1.1E + 04	1.2E + 07	5.3E + 03	5.2E + 03	8.9E + 03	5.9E + 03	5.7E + 03	4.6E + 03	6.6E + 03	5.4E + 03
	Fmean	5.1E + 03	1.9E + 04	5.4E + 06	1.5E + 09	6.2E + 03	6.0E + 03	1.6E + 04	9.3E + 03	7.6E + 03	5.9E + 03	8.7E + 03	7.0E + 03
	Std	4.2E + 02	2.7E + 04	2.0E + 07	3.3E + 09	4.9E + 02	6.1E + 02	5.4E + 03	7.1E + 03	1.5E + 03	5.8E + 02	1.3E + 03	1.0E + 03
F30	Fbest	3.3E + 07	2.1E + 08	4.7E + 08	1.7E + 10	1.0E + 08	9.3E + 07	4.5E + 08	6.8E + 07	1.2E + 08	5.2E + 07	8.7E + 07	2.2E + 07
	Fmean	6.7E + 07	9.1E + 08	1.7E + 10	7.2E + 10	2.8E + 08	1.6E + 08	2.1E + 09	1.2E + 08	1.9E + 08	1.1E + 08	2.9E + 08	4.0E + 07
	Std	2.1E + 07	9.7E + 08	1.3E + 10	3.7E + 10	1.3E + 08	6.1E + 07	1.1E + 09	3.7E + 07	5.0E + 07	4.5E + 07	1.3E + 08	1.1E + 07

		SA	GA	PSO	SMA	HHO	SSA	WOA	GWO	EBGWO
F16	best	7.5E + 03	5.0E + 03	3.1E + 03	2.7E + 03	3.8E + 03	5.7E + 03	4.8E + 03	4.8E + 03	2.9E + 03
	Mean	8.5E + 03	6.2E + 03	4.8E + 03	3.7E + 03	5.4E + 03	8.3E + 03	6.6E + 03	6.8E + 03	3.8E + 03
	Std	5.4E + 02	6.1E + 02	8.4E + 02	5.2E + 02	9.4E + 02	1.6E + 03	1.3E + 03	1.2E + 03	4.8E + 02
F17	best	6.2E + 03	3.8E + 03	3.0E + 03	**2.4E + 03**	2.8E + 03	3.7E + 03	3.5E + 03	4.2E + 03	2.6E + 03
	Mean	1.5E + 04	4.5E + 03	3.8E + 03	3.4E + 03	4.0E + 03	6.0E + 03	4.8E + 03	6.3E + 03	**3.2E + 03**
	Std	7.0E + 03	4.2E + 02	4.4E + 02	3.9E + 02	5.5E + 02	1.9E + 03	7.8E + 02	2.1E + 03	2.8E + 02
F18	best	2.9E + 07	1.9E + 07	2.0E + 06	1.3E + 06	3.8E + 06	1.1E + 07	2.2E + 06	1.7E + 07	2.6E + 05
	Mean	1.4E + 08	5.5E + 07	1.6E + 07	8.8E + 06	2.3E + 07	8.1E + 07	7.6E + 07	1.3E + 08	2.4E + 06
	Std	4.6E + 07	2.9E + 07	1.3E + 07	5.6E + 06	1.6E + 07	4.8E + 07	6.1E + 07	7.6E + 07	1.6E + 06
F19	best	1.2E + 09	9.3E + 07	**3.2E + 03**	6.9E + 03	2.4E + 05	1.0E + 07	6.4E + 06	2.6E + 08	2.5E + 04
	Mean	3.0E + 09	2.1E + 08	5.0E + 05	**3.1E + 04**	4.6E + 06	6.3E + 08	4.4E + 07	1.3E + 09	2.5E + 06
	Std	1.0E + 09	9.0E + 07	2.5E + 06	**1.6E + 04**	5.0E + 06	5.9E + 08	3.2E + 07	7.4E + 08	2.6E + 06
F20	best	4.1E + 03	3.1E + 03	3.1E + 03	2.6E + 03	2.8E + 03	3.4E + 03	3.5E + 03	3.1E + 03	2.6E + 03
	Mean	4.5E + 03	3.8E + 03	3.9E + 03	3.4E + 03	3.7E + 03	4.1E + 03	4.0E + 03	4.2E + 03	**3.1E + 03**
	Std	**1.6E + 02**	3.0E + 02	4.0E + 02	3.1E + 02	3.3E + 02	4.3E + 02	2.7E + 02	3.9E + 02	2.8E + 02
F21	best	3.1E + 03	2.9E + 03	2.6E + 03	2.5E + 03	2.8E + 03	2.8E + 03	2.9E + 03	2.9E + 03	2.6E + 03
	Mean	3.2E + 03	3.1E + 03	2.7E + 03	2.6E + 03	3.0E + 03	3.1E + 03	3.1E + 03	3.1E + 03	2.6E + 03
	Std	4.8E + 01	4.7E + 01	4.7E + 01	6.6E + 01	9.4E + 01	1.2E + 02	1.0E + 02	7.2E + 01	5.2E + 01
F22	best	1.6E + 04	1.5E + 04	1.4E + 04	8.3E + 03	1.1E + 04	1.2E + 04	1.3E + 04	1.5E + 04	**7.5E + 03**
	Mean	1.7E + 04	1.7E + 04	1.6E + 04	1.0E + 04	1.3E + 04	1.4E + 04	1.5E + 04	1.7E + 04	9.5E + 03
	Std	**3.8E + 02**	6.2E + 02	8.0E + 02	9.2E + 02	1.1E + 03	1.2E + 03	1.1E + 03	7.4E + 02	9.7E + 02
F23	best	4.1E + 03	4.0E + 03	3.0E + 03	3.0E + 03	3.7E + 03	3.8E + 03	3.6E + 03	3.6E + 03	3.0E + 03
	Mean	4.3E + 03	4.3E + 03	3.2E + 03	3.0E + 03	4.1E + 03	4.3E + 03	3.9E + 03	4.1E + 03	3.1E + 03
	Std	8.4E + 01	1.8E + 02	6.0E + 01	6.0E + 01	1.9E + 02	2.1E + 02	2.0E + 02	2.2E + 02	8.5E + 01
F24	best	4.4E + 03	4.3E + 03	3.3E + 03	3.1E + 03	3.8E + 03	4.2E + 03	3.7E + 03	3.7E + 03	3.1E + 03
	Mean	4.6E + 03	4.7E + 03	3.4E + 03	3.2E + 03	4.3E + 03	4.5E + 03	4.0E + 03	4.1E + 03	3.3E + 03
	Std	1.3E + 02	1.9E + 02	3.2E + 01	6.5E + 01	2.6E + 02	2.7E + 02	1.5E + 02	2.3E + 02	8.3E + 01
F25	best	2.6E + 04	6.9E + 03	3.3E + 03	3.1E + 03	4.5E + 03	9.4E + 03	5.1E + 03	1.0E + 04	3.1E + 03
	Mean	3.1E + 04	8.9E + 03	3.4E + 03	3.2E + 03	5.4E + 03	1.1E + 04	6.6E + 03	1.4E + 04	3.1E + 03
	Std	2.8E + 03	7.9E + 02	1.4E + 02	3.8E + 01	5.6E + 02	1.3E + 03	8.6E + 02	1.5E + 03	3.5E + 01
F26	best	1.7E + 04	1.2E + 04	7.1E + 03	3.3E + 03	1.1E + 04	1.4E + 04	1.2E + 04	1.4E + 04	3.7E + 03
	Mean	2.0E + 04	1.4E + 04	8.2E + 03	**6.3E + 03**	1.3E + 04	1.7E + 04	1.5E + 04	1.6E + 04	8.2E + 03
	Std	1.1E + 03	8.0E + 02	**5.1E + 02**	2.3E + 03	9.3E + 02	7.5E + 02	1.5E + 03	1.0E + 03	1.7E + 03
F27	best	5.5E + 03	5.3E + 03	3.6E + 03	3.4E + 03	4.4E + 03	5.7E + 03	4.0E + 03	4.7E + 03	3.5E + 03
	Mean	6.2E + 03	6.2E + 03	3.8E + 03	3.6E + 03	5.3E + 03	6.9E + 03	5.1E + 03	6.2E + 03	3.7E + 03
	Std	2.7E + 02	3.9E + 02	1.3E + 02	1.3E + 02	6.0E + 02	7.2E + 02	6.3E + 02	7.4E + 02	1.8E + 02
F28	best	1.3E + 04	7.7E + 03	3.5E + 03	3.3E + 03	4.7E + 03	8.0E + 03	5.2E + 03	9.4E + 03	3.3E + 03
	Mean	1.5E + 04	8.7E + 03	3.9E + 03	3.5E + 03	6.3E + 03	9.8E + 03	6.8E + 03	1.2E + 04	3.4E + 03
	Std	9.8E + 02	4.9E + 02	3.3E + 02	8.9E + 01	5.9E + 02	8.8E + 02	6.5E + 02	1.4E + 03	7.8E + 01
F29	best	1.2E + 04	6.8E + 03	4.6E + 03	4.4E + 03	6.3E + 03	8.4E + 03	6.8E + 03	8.1E + 03	4.4E + 03
	Mean	2.3E + 04	8.6E + 03	5.4E + 03	**5.0E + 03**	9.4E + 03	1.8E + 04	9.7E + 03	2.1E + 04	5.6E + 03
	Std	8.8E + 03	1.0E + 03	4.8E + 02	**3.5E + 02**	1.9E + 03	1.9E + 04	2.1E + 03	1.3E + 04	5.2E + 02
F30	best	2.8E + 09	3.2E + 08	1.5E + 07	**1.2E + 07**	8.4E + 07	4.8E + 08	1.1E + 08	7.9E + 08	5.3E + 07
	Mean	4.8E + 09	7.7E + 08	3.2E + 07	**1.9E + 07**	3.0E + 08	1.9E + 09	4.0E + 08	3.2E + 09	1.3E + 08
	Std	1.2E + 09	2.6E + 08	1.4E + 07	**4.4E + 06**	2.2E + 08	1.3E + 09	1.7E + 08	1.9E + 09	3.3E + 07

For 100-dimensional problems (Tables 12 and 13), EBAS with 60 random directions achieved the best results among all algorithms in five functions (F4, F7, F11, F17, and F21). In addition, EBAS got the minimum Fbest values in eight functions (F5, F8 to F10, F16, F22, F24, and F25) and got the minimum Fmean values in 11 functions (F5, F8, F10, F16, F20, F22, F24 to F27, and F29). Furthermore, the Std results of F18 and F29 were also smaller for EBAS than other algorithms. Similar to the results of 50-dimensional problems, EBGWO with 60 random directions got better results than many compared algorithms, but was still not superior to EBAS.

**Table 12:** The comparative results for 100-dimensional problems in F1-F15

		EBAS	BAS	QIBAS	BAS_ADAM	BSAS	BSO	LABAS	LCCBAS	BASGA	BASFPA	BGWO	SABAS
F1	Fbest	6.5E + 07	3.4E + 11	5.0E + 11	6.2E + 11	1.8E + 10	3.9E + 10	2.1E + 11	9.1E + 10	7.8E + 10	1.9E + 10	1.1E + 11	**1.2E + 02**
	Fmean	1.2E + 08	4.3E + 11	7.0E + 11	8.4E + 11	2.8E + 10	5.5E + 10	2.3E + 11	1.4E + 11	1.0E + 11	3.1E + 10	1.3E + 11	**1.0E + 04**
	Std	3.8E + 07	5.1E + 10	1.2E + 11	1.1E + 11	6.6E + 09	9.4E + 09	1.4E + 10	2.6E + 10	1.0E + 10	6.5E + 09	1.4E + 10	**1.5E + 04**
F3	Fbest	6.0E + 05	6.9E + 05	7.7E + 05	4.1E + 11	6.0E + 05	2.8E + 05	3.5E + 05	4.6E + 05	3.4E + 05	**2.2E + 05**	3.7E + 05	5.8E + 05
	Fmean	7.9E + 05	9.7E + 05	1.4E + 17	1.2E + 18	7.9E + 05	3.6E + 05	4.0E + 05	8.6E + 05	5.0E + 05	**3.0E + 05**	5.4E + 05	7.7E + 05
	Std	1.1E + 05	1.4E + 05	6.8E + 17	4.2E + 18	1.2E + 05	1.3E + 05	**2.9E + 04**	2.4E + 05	1.1E + 05	3.6E + 04	1.4E + 05	9.5E + 04
F4	Fbest	8.4E + 02	9.1E + 04	1.7E + 05	2.9E + 05	1.6E + 03	3.8E + 03	4.7E + 04	1.4E + 04	1.1E + 04	2.5E + 03	1.6E + 04	**7.1E + 02**
	Fmean	**1.0E + 03**	1.6E + 05	3.6E + 05	6.7E + 05	2.7E + 03	6.2E + 03	6.7E + 04	2.3E + 04	1.5E + 04	4.3E + 03	2.5E + 04	2.1E + 03
	Std	1.1E + 02	3.3E + 04	1.4E + 05	2.6E + 05	7.3E + 02	1.6E + 03	1.0E + 04	5.2E + 03	2.3E + 03	1.2E + 03	4.3E + 03	1.3E + 03
F5	Fbest	**1.0E + 03**	2.7E + 03	2.6E + 03	3.1E + 03	1.7E + 03	1.4E + 03	1.9E + 03	1.4E + 03	1.3E + 03	1.3E + 03	1.6E + 03	2.3E + 03
	Fmean	**1.3E + 03**	3.1E + 03	3.1E + 03	3.6E + 03	2.0E + 03	1.6E + 03	2.0E + 03	1.6E + 03	1.5E + 03	1.5E + 03	1.7E + 03	2.7E + 03
	Std	1.2E + 02	2.4E + 02	2.7E + 02	3.1E + 02	1.3E + 02	9.9E + 01	5.5E + 01	1.3E + 02	6.1E + 01	1.2E + 02	5.8E + 01	2.6E + 02
F6	Fbest	6.6E + 02	7.4E + 02	7.1E + 02	7.7E + 02	7.0E + 02	6.6E + 02	6.9E + 02	6.7E + 02	6.6E + 02	6.6E + 02	6.7E + 02	6.8E + 02
	Fmean	6.7E + 02	7.5E + 02	7.4E + 02	7.9E + 02	7.3E + 02	6.8E + 02	7.0E + 02	6.8E + 02	6.7E + 02	6.8E + 02	6.8E + 02	7.1E + 02
	Std	7.6E + 00	1.1E + 01	1.5E + 01	1.3E + 01	1.9E + 01	8.1E + 00	4.3E + 00	6.9E + 00	4.0E + 00	6.1E + 00	5.3E + 00	2.1E + 01
F7	Fbest	**1.6E + 03**	9.6E + 03	1.2E + 04	1.4E + 04	2.6E + 03	2.4E + 03	3.6E + 03	4.1E + 03	2.7E + 03	2.2E + 03	3.2E + 03	4.0E + 03
	Fmean	**1.8E + 03**	1.2E + 04	1.5E + 04	1.6E + 04	2.9E + 03	2.8E + 03	4.1E + 03	5.4E + 03	3.4E + 03	2.7E + 03	3.5E + 03	5.4E + 03
	Std	1.4E + 02	1.2E + 03	1.5E + 03	1.5E + 03	1.7E + 02	2.0E + 02	2.1E + 02	6.3E + 02	3.1E + 02	2.9E + 02	1.4E + 02	8.2E + 02
F8	Fbest	**1.4E + 03**	3.2E + 03	3.0E + 03	3.4E + 03	2.0E + 03	1.7E + 03	2.3E + 03	1.8E + 03	1.7E + 03	1.6E + 03	2.0E + 03	2.8E + 03
	Fmean	**1.6E + 03**	3.6E + 03	3.7E + 03	4.3E + 03	2.3E + 03	1.9E + 03	2.5E + 03	2.0E + 03	1.8E + 03	1.9E + 03	2.2E + 03	3.1E + 03
	Std	1.2E + 02	2.5E + 02	3.8E + 02	3.2E + 02	1.4E + 02	9.0E + 01	7.1E + 01	1.5E + 02	7.3E + 01	1.5E + 02	6.9E + 01	2.3E + 02
F9	Fbest	2.9E + 04	1.3E + 05	7.2E + 04	1.8E + 05	1.2E + 05	3.4E + 04	6.5E + 04	3.3E + 04	2.8E + 04	3.0E + 04	3.9E + 04	4.4E + 04
	Fmean	3.8E + 04	1.7E + 05	1.3E + 05	2.4E + 05	1.8E + 05	4.7E + 04	7.6E + 04	4.5E + 04	3.5E + 04	4.4E + 04	4.5E + 04	5.8E + 04
	Std	6.4E + 03	2.3E + 04	4.5E + 04	3.3E + 04	3.6E + 04	9.8E + 03	6.9E + 03	7.5E + 03	4.1E + 03	8.9E + 03	4.9E + 03	6.9E + 03
F10	Fbest	**1.4E + 04**	2.8E + 04	2.5E + 04	3.6E + 04	2.6E + 04	2.8E + 04	3.1E + 04	1.7E + 04	1.6E + 04	1.9E + 04	2.3E + 04	1.5E + 04
	Fmean	**1.6E + 04**	3.1E + 04	3.1E + 04	3.8E + 04	3.0E + 04	3.1E + 04	3.2E + 04	2.0E + 04	1.9E + 04	2.1E + 04	2.6E + 04	1.8E + 04
	Std	1.2E + 03	1.7E + 03	3.2E + 03	1.1E + 03	1.8E + 03	1.7E + 03	**7.0E + 02**	3.2E + 03	1.7E + 03	1.5E + 03	1.4E + 03	2.0E + 03
F11	Fbest	1.6E + 04	3.6E + 05	2.9E + 05	2.3E + 10	3.3E + 04	1.1E + 05	1.1E + 05	6.8E + 04	1.1E + 05	2.4E + 04	1.5E + 05	**5.9E + 03**
	Fmean	2.9E + 04	6.0E + 05	4.2E + 12	2.5E + 14	7.3E + 04	1.6E + 05	1.5E + 05	1.4E + 05	2.3E + 05	4.0E + 04	2.5E + 05	**1.6E + 04**
	Std	1.0E + 04	1.4E + 05	1.9E + 13	6.8E + 14	2.3E + 04	2.6E + 04	2.2E + 04	1.0E + 05	7.8E + 04	1.1E + 04	5.3E + 04	7.1E + 03
F12	Fbest	1.8E + 08	7.0E + 10	2.3E + 11	2.9E + 11	3.3E + 09	3.1E + 09	8.6E + 10	2.1E + 10	1.0E + 10	1.2E + 09	2.7E + 10	**3.5E + 07**
	Fmean	6.7E + 08	1.9E + 11	4.1E + 11	6.2E + 11	5.8E + 09	6.0E + 09	1.2E + 11	3.8E + 10	2.4E + 10	2.8E + 09	4.1E + 10	**1.5E + 08**
	Std	2.8E + 08	5.2E + 10	1.1E + 11	1.4E + 11	1.8E + 09	1.5E + 09	1.4E + 10	1.1E + 10	6.1E + 09	7.6E + 08	8.7E + 09	**5.9E + 07**
F13	Fbest	1.0E + 05	1.8E + 10	1.1E + 10	8.2E + 10	2.2E + 08	6.0E + 07	1.9E + 10	1.2E + 08	1.4E + 08	**2.7E + 04**	8.1E + 08	4.7E + 04
	Fmean	1.6E + 05	3.9E + 10	5.5E + 10	1.6E + 11	6.3E + 08	1.6E + 08	2.7E + 10	1.7E + 09	1.4E + 09	5.0E + 05	2.8E + 09	1.0E + 05
	Std	3.8E + 04	1.4E + 10	2.5E + 10	3.0E + 10	2.6E + 08	6.6E + 07	3.9E + 09	9.2E + 08	1.1E + 09	1.8E + 06	1.7E + 09	4.5E + 04
F14	Fbest	3.4E + 05	2.0E + 07	5.8E + 06	1.6E + 09	2.7E + 06	8.3E + 05	1.5E + 07	1.6E + 06	4.7E + 06	7.1E + 05	1.2E + 07	**2.3E + 05**
	Fmean	1.8E + 06	1.2E + 08	8.8E + 08	1.0E + 10	1.1E + 07	4.7E + 06	4.6E + 07	7.5E + 06	1.5E + 07	2.8E + 06	2.2E + 07	**8.1E + 05**
	Std	8.0E + 05	7.8E + 07	9.1E + 08	8.9E + 09	4.8E + 06	3.2E + 06	1.8E + 07	3.8E + 06	6.9E + 06	2.0E + 06	7.4E + 06	**4.3E + 05**
F15	Fbest	4.8E + 04	3.5E + 09	3.9E + 06	5.9E + 10	7.9E + 07	4.0E + 06	5.9E + 09	2.4E + 05	1.6E + 06	2.4E + 04	7.6E + 05	**2.3E + 04**
	Fmean	1.2E + 05	1.3E + 10	1.3E + 10	1.0E + 11	2.0E + 08	1.7E + 07	1.1E + 10	1.0E + 07	5.6E + 07	7.4E + 04	1.4E + 08	9.8E + 04
	Std	4.3E + 04	8.8E + 09	1.2E + 10	2.4E + 10	8.0E + 07	9.0E + 06	3.4E + 09	2.4E + 07	1.3E + 08	2.9E + 04	1.7E + 08	4.6E + 04

		SA	GA	PSO	SMA	HHO	SSA	WOA	GWO	EBGWO
F1	Fbest	4.3E + 11	1.7E + 11	2.1E + 10	1.0E + 09	9.6E + 10	2.1E + 11	1.1E + 11	2.2E + 11	5.0E + 07
	Fmean	4.7E + 11	2.0E + 11	3.2E + 10	2.1E + 09	1.2E + 11	2.4E + 11	1.4E + 11	2.5E + 11	7.8E + 07
	Std	1.9E + 10	1.3E + 10	5.3E + 09	5.4E + 08	1.0E + 10	1.2E + 10	1.6E + 10	1.5E + 10	1.5E + 07
F3	Fbest	6.1E + 05	5.0E + 05	5.7E + 05	3.1E + 05	3.2E + 05	3.7E + 05	6.4E + 05	4.0E + 05	3.3E + 05
	Fmean	7.2E + 05	7.8E + 05	7.8E + 05	8.2E + 05	4.7E + 05	5.6E + 05	9.6E + 05	6.0E + 05	4.8E + 05
	Std	5.6E + 04	2.2E + 05	1.2E + 05	4.1E + 05	1.9E + 05	1.7E + 05	1.6E + 05	1.5E + 05	1.2E + 05
F4	Fbest	1.3E + 05	3.8E + 04	2.9E + 03	1.0E + 03	1.8E + 04	5.7E + 04	1.9E + 04	6.0E + 04	8.9E + 02
	Fmean	1.6E + 05	4.8E + 04	3.9E + 03	1.2E + 03	2.6E + 04	7.8E + 04	2.9E + 04	8.3E + 04	1.0E + 03
	Std	1.4E + 04	5.0E + 03	9.3E + 02	1.6E + 02	5.2E + 03	9.9E + 03	5.5E + 03	1.4E + 04	**7.5E + 01**
F5	Fbest	2.6E + 03	1.9E + 03	1.5E + 03	1.3E + 03	1.6E + 03	1.8E + 03	1.8E + 03	1.8E + 03	1.2E + 03
	Fmean	2.7E + 03	2.0E + 03	1.7E + 03	1.4E + 03	1.7E + 03	2.0E + 03	2.0E + 03	2.1E + 03	1.3E + 03
	Std	5.7E + 01	**4.5E + 01**	8.1E + 01	9.9E + 01	5.6E + 01	7.5E + 01	1.4E + 02	7.0E + 01	5.7E + 01
F6	Fbest	7.3E + 02	7.0E + 02	**6.4E + 02**	6.5E + 02	6.8E + 02	6.9E + 02	6.9E + 02	6.9E + 02	6.6E + 02
	Fmean	7.4E + 02	7.1E + 02	**6.6E + 02**	6.7E + 02	7.0E + 02	7.1E + 02	7.1E + 02	7.1E + 02	6.7E + 02
	Std	4.0E + 00	**3.9E + 00**	8.5E + 00	6.7E + 00	4.3E + 00	6.6E + 00	1.0E + 01	5.6E + 00	4.5E + 00
F7	Fbest	9.7E + 03	3.4E + 03	2.8E + 03	2.0E + 03	3.6E + 03	3.6E + 03	3.5E + 03	3.7E + 03	1.8E + 03
	Fmean	1.1E + 04	3.7E + 03	3.2E + 03	2.4E + 03	3.8E + 03	3.9E + 03	3.8E + 03	3.9E + 03	2.2E + 03
	Std	3.8E + 02	1.5E + 02	2.2E + 02	2.0E + 02	1.1E + 02	1.1E + 02	1.4E + 02	**1.0E + 02**	2.9E + 02
F8	Fbest	2.8E + 03	2.4E + 03	1.7E + 03	1.6E + 03	2.1E + 03	2.3E + 03	2.1E + 03	2.3E + 03	1.5E + 03
	Fmean	3.1E + 03	2.5E + 03	1.9E + 03	1.8E + 03	2.2E + 03	2.5E + 03	2.4E + 03	2.5E + 03	1.7E + 03
	Std	7.5E + 01	**5.4E + 01**	9.7E + 01	1.1E + 02	5.5E + 01	8.3E + 01	1.1E + 02	8.0E + 01	8.8E + 01
F9	Fbest	1.4E + 05	5.5E + 04	2.6E + 04	3.7E + 04	6.2E + 04	4.8E + 04	5.7E + 04	6.1E + 04	**2.4E + 04**
	Fmean	1.6E + 05	7.2E + 04	5.3E + 04	4.5E + 04	7.0E + 04	6.1E + 04	9.1E + 04	7.7E + 04	**2.7E + 04**
	Std	1.1E + 04	7.5E + 03	2.1E + 04	5.6E + 03	4.6E + 03	5.5E + 03	2.4E + 04	6.6E + 03	**1.6E + 03**
F10	Fbest	3.0E + 04	2.9E + 04	2.8E + 04	1.8E + 04	2.3E + 04	2.3E + 04	2.6E + 04	3.0E + 04	1.5E + 04
	Fmean	3.3E + 04	3.2E + 04	3.2E + 04	2.1E + 04	2.6E + 04	2.7E + 04	3.0E + 04	3.2E + 04	1.7E + 04
	Std	9.2E + 02	9.1E + 02	1.4E + 03	1.3E + 03	1.7E + 03	2.3E + 03	1.6E + 03	9.3E + 02	1.3E + 03
F11	Fbest	2.1E + 05	1.4E + 05	9.5E + 04	2.8E + 04	1.2E + 05	1.7E + 05	2.1E + 05	1.6E + 05	9.1E + 03
	Fmean	3.4E + 05	2.4E + 05	2.0E + 05	6.1E + 04	1.9E + 05	3.5E + 05	3.6E + 05	2.7E + 05	1.7E + 04
	Std	4.7E + 04	6.1E + 04	5.1E + 04	1.7E + 04	4.2E + 04	1.4E + 05	1.4E + 05	6.5E + 04	**6.3E + 03**
F12	Fbest	1.8E + 11	7.7E + 10	3.1E + 09	1.7E + 08	2.5E + 10	1.3E + 11	2.7E + 10	1.3E + 11	1.5E + 08
	Fmean	2.2E + 11	1.1E + 11	5.5E + 09	6.2E + 08	5.0E + 10	1.6E + 11	4.7E + 10	1.6E + 11	6.0E + 08
	Std	1.7E + 10	1.5E + 10	2.1E + 09	2.5E + 08	1.2E + 10	1.7E + 10	1.1E + 10	2.1E + 10	3.1E + 08
F13	Fbest	3.8E + 10	1.3E + 10	2.4E + 07	4.7E + 05	2.4E + 09	2.5E + 10	2.7E + 09	1.9E + 10	7.2E + 04
	Fmean	5.0E + 10	1.9E + 10	1.1E + 08	6.1E + 06	6.0E + 09	3.4E + 10	5.1E + 09	3.6E + 10	**1.0E + 05**
	Std	5.3E + 09	3.5E + 09	6.9E + 07	1.2E + 07	1.8E + 09	5.2E + 09	1.5E + 09	7.0E + 09	**1.9E + 04**
F14	Fbest	6.5E + 07	1.8E + 07	3.2E + 06	3.0E + 06	4.0E + 06	1.5E + 07	4.1E + 06	2.2E + 07	9.0E + 05
	Fmean	1.7E + 08	4.1E + 07	2.0E + 07	8.4E + 06	1.6E + 07	3.2E + 07	2.6E + 07	6.5E + 07	3.1E + 06
	Std	4.7E + 07	1.7E + 07	9.9E + 06	5.0E + 06	6.9E + 06	1.2E + 07	1.4E + 07	3.2E + 07	1.5E + 06
F15	Fbest	1.6E + 10	2.1E + 09	1.6E + 06	6.2E + 04	1.7E + 08	4.5E + 09	2.8E + 08	6.0E + 09	4.0E + 04
	Fmean	2.2E + 10	4.9E + 09	4.3E + 07	1.5E + 06	9.3E + 08	1.4E + 10	1.0E + 09	1.6E + 10	**7.2E + 04**
	Std	3.1E + 09	1.6E + 09	7.9E + 07	4.0E + 06	6.8E + 08	4.3E + 09	4.6E + 08	5.0E + 09	**2.8E + 04**

**Table 13:** The comparative results for 100-dimensional problems in F16-F30

		EBAS	BAS	QIBAS	BAS_ADAM	BSAS	BSO	LABAS	LCCBAS	BASGA	BASFPA	BGWO	SABAS
F16	Fbest	**4.5E + 03**	1.5E + 04	2.1E + 04	4.7E + 04	7.1E + 03	6.3E + 03	1.5E + 04	8.8E + 03	7.2E + 03	7.0E + 03	1.0E + 04	6.4E + 03
	Fmean	**6.2E + 03**	2.4E + 04	5.1E + 04	1.0E + 05	1.0E + 04	8.1E + 03	1.7E + 04	1.1E + 04	9.7E + 03	8.6E + 03	1.3E + 04	9.2E + 03
	Std	8.5E + 02	5.8E + 03	1.8E + 04	4.7E + 04	1.3E + 03	**7.1E + 02**	1.2E + 03	1.8E + 03	1.1E + 03	9.7E + 02	1.5E + 03	1.7E + 03
F17	Fbest	**4.4E + 03**	4.0E + 04	1.4E + 05	5.9E + 07	6.9E + 03	4.6E + 03	6.8E + 04	5.5E + 03	5.4E + 03	5.0E + 03	6.6E + 03	5.5E + 03
	Fmean	**5.3E + 03**	5.4E + 06	1.2E + 08	1.1E + 10	8.2E + 03	5.9E + 03	5.3E + 05	7.1E + 03	7.9E + 03	6.1E + 03	1.6E + 04	6.8E + 03
	Std	5.2E + 02	1.2E + 07	2.2E + 08	2.2E + 10	6.2E + 02	**4.8E + 02**	7.1E + 05	7.8E + 02	1.7E + 03	6.8E + 02	1.7E + 04	1.2E + 03
F18	Fbest	9.7E + 05	2.4E + 07	6.7E + 07	1.2E + 09	7.1E + 06	1.2E + 06	2.7E + 07	2.0E + 06	2.6E + 06	1.2E + 06	6.0E + 06	**2.2E + 05**
	Fmean	3.3E + 06	1.7E + 08	2.5E + 09	9.4E + 09	1.6E + 07	4.6E + 06	7.0E + 07	5.9E + 06	1.1E + 07	3.1E + 06	2.0E + 07	**1.7E + 06**
	Std	2.1E + 06	1.5E + 08	3.2E + 09	7.3E + 09	8.4E + 06	2.2E + 06	3.1E + 07	3.2E + 06	6.6E + 06	1.9E + 06	9.8E + 06	**1.3E + 06**
F19	Fbest	1.5E + 06	2.6E + 09	1.2E + 07	5.7E + 10	1.1E + 08	9.4E + 06	6.7E + 09	1.8E + 06	4.0E + 06	2.0E + 06	1.0E + 07	8.7E + 05
	Fmean	1.6E + 07	1.3E + 10	2.6E + 10	1.1E + 11	2.3E + 08	3.8E + 07	1.1E + 10	8.7E + 07	5.6E + 07	2.4E + 07	2.1E + 08	4.3E + 06
	Std	8.5E + 06	7.0E + 09	1.5E + 10	2.8E + 10	9.5E + 07	2.5E + 07	2.2E + 09	9.5E + 07	6.5E + 07	1.9E + 07	2.0E + 08	1.7E + 06
F20	Fbest	**4.1E + 03**	6.7E + 03	6.1E + 03	9.2E + 03	5.9E + 03	5.0E + 03	7.1E + 03	5.2E + 03	4.5E + 03	4.6E + 03	5.1E + 03	5.2E + 03
	Fmean	**5.2E + 03**	7.9E + 03	7.9E + 03	1.1E + 04	7.6E + 03	6.3E + 03	7.9E + 03	6.5E + 03	5.4E + 03	5.8E + 03	6.0E + 03	6.6E + 03
	Std	5.7E + 02	5.3E + 02	8.5E + 02	5.4E + 02	6.5E + 02	6.4E + 02	2.7E + 02	7.5E + 02	4.6E + 02	4.7E + 02	5.0E + 02	9.6E + 02
F21	Fbest	**2.9E + 03**	4.5E + 03	5.2E + 03	5.5E + 03	3.6E + 03	3.2E + 03	4.2E + 03	3.7E + 03	3.1E + 03	3.2E + 03	3.8E + 03	3.9E + 03
	Fmean	**3.1E + 03**	5.3E + 03	6.3E + 03	7.7E + 03	3.8E + 03	3.4E + 03	4.5E + 03	4.2E + 03	3.3E + 03	3.5E + 03	4.1E + 03	4.6E + 03
	Std	1.2E + 02	3.5E + 02	1.1E + 03	1.9E + 03	1.5E + 02	1.2E + 02	1.3E + 02	2.6E + 02	1.2E + 02	2.1E + 02	1.7E + 02	3.4E + 02
F22	Fbest	1.6E + 04	2.9E + 04	2.9E + 04	3.8E + 04	2.9E + 04	2.9E + 04	3.4E + 04	1.7E + 04	2.1E + 04	2.1E + 04	2.5E + 04	1.7E + 04
	Fmean	**1.9E + 04**	3.4E + 04	3.4E + 04	4.1E + 04	3.3E + 04	3.4E + 04	3.5E + 04	2.1E + 04	2.4E + 04	2.4E + 04	2.8E + 04	2.2E + 04
	Std	1.3E + 03	1.8E + 03	2.4E + 03	1.6E + 03	1.7E + 03	1.7E + 03	**5.6E + 02**	1.6E + 03	1.2E + 03	2.1E + 03	1.2E + 03	2.5E + 03
F23	Fbest	3.6E + 03	5.8E + 03	6.5E + 03	8.6E + 03	4.1E + 03	4.2E + 03	5.6E + 03	5.0E + 03	3.9E + 03	4.1E + 03	4.7E + 03	4.8E + 03
	Fmean	3.8E + 03	8.0E + 03	8.7E + 03	1.3E + 04	4.4E + 03	4.7E + 03	6.0E + 03	5.9E + 03	4.1E + 03	4.6E + 03	5.4E + 03	6.5E + 03
	Std	1.2E + 02	1.4E + 03	2.0E + 03	2.5E + 03	1.6E + 02	3.4E + 02	3.0E + 02	5.1E + 02	1.3E + 02	2.8E + 02	3.8E + 02	1.2E + 03
F24	Fbest	**3.9E + 03**	8.0E + 03	9.3E + 03	1.4E + 04	4.6E + 03	5.6E + 03	8.5E + 03	8.1E + 03	4.9E + 03	5.2E + 03	6.3E + 03	5.1E + 03
	Fmean	**4.2E + 03**	1.1E + 04	1.5E + 04	2.3E + 04	4.8E + 03	6.4E + 03	1.0E + 04	1.0E + 04	5.9E + 03	5.8E + 03	7.4E + 03	5.9E + 03
	Std	1.3E + 02	1.5E + 03	3.7E + 03	4.3E + 03	1.8E + 02	5.5E + 02	9.5E + 02	1.0E + 03	3.7E + 02	3.8E + 02	6.1E + 02	5.2E + 02
F25	Fbest	3.5E + 03	4.2E + 04	8.4E + 04	1.7E + 05	4.8E + 03	6.3E + 03	2.0E + 04	8.3E + 03	8.2E + 03	4.8E + 03	1.0E + 04	**3.5E + 03**
	Fmean	3.8E + 03	7.2E + 04	1.5E + 05	2.9E + 05	5.7E + 03	7.7E + 03	2.4E + 04	1.3E + 04	1.1E + 04	5.7E + 03	1.3E + 04	5.0E + 03
	Std	1.2E + 02	2.0E + 04	4.0E + 04	8.2E + 04	4.6E + 02	8.2E + 02	1.6E + 03	2.1E + 03	1.2E + 03	5.1E + 02	1.3E + 03	1.3E + 03
F26	Fbest	1.3E + 04	5.0E + 04	7.8E + 04	1.1E + 05	1.9E + 04	2.0E + 04	3.7E + 04	3.5E + 04	2.6E + 04	1.7E + 04	3.2E + 04	**3.4E + 03**
	Fmean	**1.6E + 04**	6.7E + 04	1.3E + 05	1.7E + 05	2.2E + 04	2.8E + 04	4.3E + 04	4.6E + 04	3.3E + 04	2.5E + 04	3.7E + 04	3.3E + 04
	Std	1.3E + 03	9.5E + 03	3.0E + 04	5.3E + 04	1.6E + 03	4.3E + 03	2.7E + 03	9.7E + 03	2.8E + 03	2.9E + 03	3.3E + 03	7.2E + 03
F27	Fbest	3.6E + 03	8.6E + 03	**3.2E + 03**	1.5E + 04	3.7E + 03	4.2E + 03	9.1E + 03	5.7E + 03	5.1E + 03	3.9E + 03	5.1E + 03	3.9E + 03
	Fmean	3.9E + 03	1.2E + 04	1.3E + 04	3.0E + 04	4.1E + 03	5.3E + 03	1.2E + 04	9.3E + 03	6.2E + 03	4.7E + 03	6.9E + 03	5.7E + 03
	Std	1.3E + 02	2.6E + 03	8.8E + 03	7.1E + 03	1.7E + 02	1.2E + 03	1.1E + 03	3.1E + 03	5.4E + 02	3.9E + 02	8.8E + 02	1.8E + 03
F28	Fbest	3.7E + 03	2.9E + 04	**3.3E + 03**	8.1E + 04	4.7E + 03	8.5E + 03	2.2E + 04	1.4E + 04	1.1E + 04	6.7E + 03	1.4E + 04	3.5E + 03
	Fmean	4.2E + 03	4.5E + 04	2.1E + 04	1.2E + 05	5.8E + 03	1.2E + 04	2.7E + 04	2.1E + 04	1.4E + 04	8.5E + 03	1.7E + 04	5.5E + 03
	Std	3.3E + 02	8.2E + 03	3.4E + 04	2.6E + 04	9.6E + 02	5.8E + 03	2.1E + 03	1.1E + 04	1.5E + 03	1.2E + 03	1.3E + 03	2.8E + 03
F29	Fbest	7.4E + 03	3.1E + 04	5.8E + 05	2.9E + 07	1.1E + 04	9.0E + 03	3.1E + 04	1.1E + 04	1.1E + 04	1.0E + 04	1.5E + 04	9.2E + 03
	Fmean	9.0E + 03	1.4E + 06	3.1E + 07	1.7E + 09	1.2E + 04	1.2E + 04	6.5E + 04	1.6E + 04	1.6E + 04	1.2E + 04	2.0E + 04	1.3E + 04
	Std	7.1E + 02	1.9E + 06	9.8E + 07	2.7E + 09	1.0E + 03	1.4E + 03	2.6E + 04	2.4E + 03	3.6E + 03	1.3E + 03	3.8E + 03	2.4E + 03
F30	Fbest	5.5E + 07	1.5E + 10	3.8E + 10	8.6E + 10	3.5E + 08	4.2E + 08	1.6E + 10	3.5E + 08	6.2E + 08	2.2E + 08	6.1E + 08	**9.3E + 06**
	Fmean	1.2E + 08	3.3E + 10	8.0E + 10	1.7E + 11	7.6E + 08	7.5E + 08	2.3E + 10	1.4E + 09	2.1E + 09	4.6E + 08	2.9E + 09	3.7E + 07
	Std	4.5E + 07	1.2E + 10	2.9E + 10	4.5E + 10	3.1E + 08	2.3E + 08	3.9E + 09	9.0E + 08	9.8E + 08	2.3E + 08	1.6E + 09	2.3E + 07

		SA	GA	PSO	SMA	HHO	SSA	WOA	GWO	EBGWO
F16	Fbest	1.8E + 04	1.3E + 04	9.7E + 03	5.6E + 03	1.0E + 04	1.7E + 04	1.2E + 04	1.5E + 04	6.6E + 03
	Fmean	2.3E + 04	1.6E + 04	1.1E + 04	6.7E + 03	1.3E + 04	2.3E + 04	1.7E + 04	2.0E + 04	7.8E + 03
	Std	1.6E + 03	1.6E + 03	8.6E + 02	8.4E + 02	1.6E + 03	3.6E + 03	2.3E + 03	2.8E + 03	7.2E + 02
F17	Fbest	5.5E + 05	1.4E + 04	6.2E + 03	4.6E + 03	8.7E + 03	9.0E + 04	9.6E + 03	6.0E + 04	4.7E + 03
	Fmean	3.4E + 06	7.3E + 04	7.7E + 03	5.8E + 03	2.6E + 04	1.7E + 06	9.8E + 04	1.3E + 06	5.6E + 03
	Std	2.1E + 06	8.3E + 04	6.4E + 02	7.3E + 02	2.4E + 04	2.3E + 06	1.8E + 05	1.4E + 06	5.6E + 02
F18	Fbest	1.6E + 08	2.3E + 07	8.8E + 06	8.3E + 06	4.7E + 06	1.4E + 07	7.6E + 06	1.8E + 07	1.1E + 06
	Fmean	3.0E + 08	6.1E + 07	2.9E + 07	1.4E + 07	1.5E + 07	6.0E + 07	2.6E + 07	8.9E + 07	3.4E + 06
	Std	8.0E + 07	2.4E + 07	1.4E + 07	4.8E + 06	7.0E + 06	4.0E + 07	1.2E + 07	5.4E + 07	1.6E + 06
F19	Fbest	1.3E + 10	3.1E + 09	2.7E + 06	**2.9E + 05**	9.1E + 07	4.3E + 09	5.0E + 08	7.3E + 09	9.3E + 05
	Fmean	2.2E + 10	5.9E + 09	2.0E + 07	**1.4E + 06**	9.1E + 08	1.3E + 10	9.5E + 08	1.5E + 10	1.4E + 07
	Std	3.5E + 09	1.6E + 09	2.5E + 07	**8.4E + 05**	6.2E + 08	4.3E + 09	3.6E + 08	4.6E + 09	1.0E + 07
F20	Fbest	7.3E + 03	6.4E + 03	6.6E + 03	4.9E + 03	4.8E + 03	5.8E + 03	6.5E + 03	6.3E + 03	4.5E + 03
	Fmean	8.2E + 03	7.5E + 03	7.8E + 03	6.0E + 03	6.4E + 03	7.0E + 03	7.4E + 03	7.6E + 03	5.4E + 03
	Std	**2.4E + 02**	4.3E + 02	4.6E + 02	6.2E + 02	5.3E + 02	5.6E + 02	4.8E + 02	5.8E + 02	4.8E + 02
F21	Fbest	4.5E + 03	4.3E + 03	3.3E + 03	3.1E + 03	4.0E + 03	4.3E + 03	4.0E + 03	4.2E + 03	3.0E + 03
	Fmean	4.8E + 03	4.5E + 03	3.5E + 03	3.3E + 03	4.4E + 03	4.6E + 03	4.5E + 03	4.5E + 03	3.3E + 03
	Std	1.1E + 02	1.0E + 02	**7.6E + 01**	1.1E + 02	1.9E + 02	2.3E + 02	1.9E + 02	1.7E + 02	1.3E + 02
F22	Fbest	3.4E + 04	3.2E + 04	3.2E + 04	2.0E + 04	2.7E + 04	2.5E + 04	3.1E + 04	3.3E + 04	**1.5E + 04**
	Fmean	3.6E + 04	3.4E + 04	3.5E + 04	2.2E + 04	2.9E + 04	2.9E + 04	3.3E + 04	3.5E + 04	2.0E + 04
	Std	5.7E + 02	8.0E + 02	9.9E + 02	1.5E + 03	1.2E + 03	1.7E + 03	1.3E + 03	9.5E + 02	1.6E + 03
F23	Fbest	6.1E + 03	6.0E + 03	3.7E + 03	**3.5E + 03**	5.2E + 03	5.4E + 03	4.9E + 03	5.1E + 03	3.9E + 03
	Fmean	6.5E + 03	6.8E + 03	3.9E + 03	**3.7E + 03**	6.0E + 03	6.3E + 03	5.4E + 03	5.8E + 03	4.3E + 03
	Std	2.0E + 02	3.5E + 02	**9.8E + 01**	1.2E + 02	6.1E + 02	4.9E + 02	2.2E + 02	4.8E + 02	3.2E + 02
F24	Fbest	9.0E + 03	7.3E + 03	4.4E + 03	4.0E + 03	7.4E + 03	7.8E + 03	6.3E + 03	7.4E + 03	4.2E + 03
	Fmean	1.0E + 04	1.1E + 04	4.5E + 03	4.3E + 03	8.8E + 03	9.5E + 03	7.1E + 03	8.6E + 03	4.7E + 03
	Std	5.2E + 02	1.1E + 03	**6.6E + 01**	1.4E + 02	8.0E + 02	6.7E + 02	4.0E + 02	7.5E + 02	3.2E + 02
F25	Fbest	7.2E + 04	1.6E + 04	6.1E + 03	3.7E + 03	9.6E + 03	1.9E + 04	1.0E + 04	2.2E + 04	3.5E + 03
	Fmean	8.3E + 04	1.8E + 04	7.7E + 03	3.9E + 03	1.1E + 04	2.3E + 04	1.4E + 04	2.7E + 04	**3.7E + 03**
	Std	5.4E + 03	1.1E + 03	1.2E + 03	1.4E + 02	1.0E + 03	2.1E + 03	1.4E + 03	2.6E + 03	**1.0E + 02**
F26	Fbest	5.3E + 04	3.9E + 04	1.6E + 04	1.4E + 04	3.1E + 04	4.5E + 04	3.3E + 04	3.9E + 04	1.6E + 04
	Fmean	6.3E + 04	4.3E + 04	1.8E + 04	1.7E + 04	3.5E + 04	4.9E + 04	4.0E + 04	4.8E + 04	2.0E + 04
	Std	4.3E + 03	2.1E + 03	**9.6E + 02**	2.0E + 03	1.9E + 03	2.2E + 03	4.1E + 03	3.6E + 03	2.2E + 03
F27	Fbest	1.0E + 04	1.0E + 04	4.0E + 03	3.6E + 03	5.7E + 03	1.1E + 04	4.8E + 03	8.7E + 03	3.7E + 03
	Fmean	1.2E + 04	1.1E + 04	4.4E + 03	**3.8E + 03**	8.3E + 03	1.4E + 04	6.2E + 03	1.1E + 04	4.1E + 03
	Std	6.1E + 02	8.1E + 02	1.9E + 02	**1.2E + 02**	1.4E + 03	1.7E + 03	9.4E + 02	1.4E + 03	2.2E + 02
F28	Fbest	4.7E + 04	2.3E + 04	6.4E + 03	3.8E + 03	1.1E + 04	2.4E + 04	1.5E + 04	2.2E + 04	3.6E + 03
	Fmean	5.3E + 04	2.6E + 04	9.6E + 03	4.1E + 03	1.4E + 04	2.6E + 04	1.8E + 04	2.8E + 04	**3.7E + 03**
	Std	2.6E + 03	1.2E + 03	1.9E + 03	2.0E + 02	9.7E + 02	9.6E + 02	1.5E + 03	2.3E + 03	**6.1E + 01**
F29	Fbest	2.1E + 05	1.7E + 04	9.7E + 03	**7.1E + 03**	1.1E + 04	3.2E + 04	1.6E + 04	3.1E + 04	8.5E + 03
	Fmean	6.3E + 05	2.5E + 04	1.1E + 04	**8.4E + 03**	1.7E + 04	1.5E + 05	2.4E + 04	1.9E + 05	1.0E + 04
	Std	2.6E + 05	6.4E + 03	6.7E + 02	**6.6E + 02**	3.1E + 03	1.0E + 05	5.6E + 03	1.8E + 05	9.1E + 02
F30	Fbest	2.6E + 10	1.1E + 10	2.6E + 07	9.4E + 06	2.2E + 09	9.9E + 09	2.4E + 09	1.6E + 10	8.3E + 07
	Fmean	3.6E + 10	1.5E + 10	8.8E + 07	**3.3E + 07**	4.9E + 09	2.8E + 10	4.7E + 09	3.0E + 10	2.0E + 08
	Std	4.8E + 09	3.2E + 09	6.6E + 07	**1.8E + 07**	1.6E + 09	7.1E + 09	1.4E + 09	7.8E + 09	1.0E + 08

Table 14 shows the average running time of all algorithms. The running time required by EBAS is in the middle range of all algorithms. Although it is not as fast as BAS, it is not as slow as BASGA or LCCBAS. EBAS with 30 (or 60) random directions requires less running time in 50-dimensional (or 60-dimensional) problems than 12 (or 6) comparison algorithms, but still finds better optimization results, indicating that EBAS has high optimization efficiency. As mentioned previously, fewer random directions can be used to reduce the running time of EBAS. Of course, the decrease in random directions will reduce the goodness of the optimal solution. However, as proved by the previous analysis results, EBAS with only two random directions is already capable to greatly improve the optimization efficiency of the BAS algorithm. In all, the success of EBAS not only comes from the improved optimization efficiency, but also attributes to the high degree of freedom when it is utilized. Indeed, the running time of EBAS can be finely controlled according to the actual requirements, and it can also be easily integrated with other algorithms for its simplicity.

**Table 14:** The average running time (seconds) of algorithms

N = 50	EBAS	BAS	QIBAS	BAS_ADAM	BSAS	BSO	LABAS	LCCBAS	BASGA	BASFPA
	0.33	0.02	0.02	0.06	0.22	0.59	0.81	1.83	7.10	0.96
BGWO	SABAS	SA	GA	PSO	SMA	HHO	SSA	WOA	GWO	EBGWO
0.22	1.21	0.83	0.62	0.20	0.89	0.48	0.39	0.20	0.20	0.54

N = 100	EBAS	BAS	QIBAS	BAS_ADAM	BSAS	BSO	LABAS	LCCBAS	BASGA	BASFPA
	2.32	0.07	0.09	0.27	0.76	1.99	2.31	5.21	21.38	2.89
BGWO	SABAS	SA	GA	PSO	SMA	HHO	SSA	WOA	GWO	EBGWO
0.71	3.78	2.53	1.93	0.68	1.95	1.56	1.10	0.65	0.66	3.12

### Convergence curve of the algorithms

**Fig. 3:**
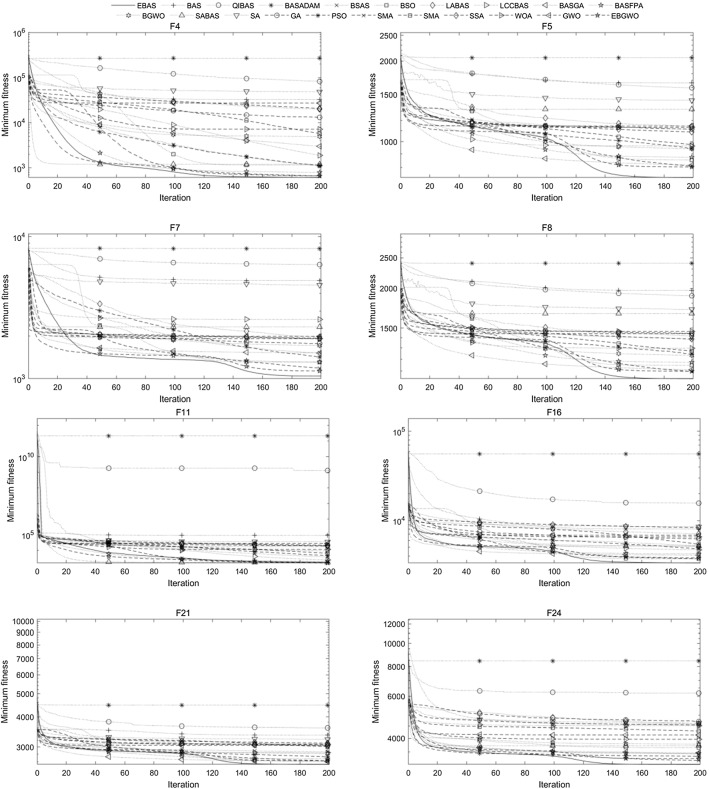
Convergence curve of partial functions for 50-dimensional problems

**Fig. 4:**
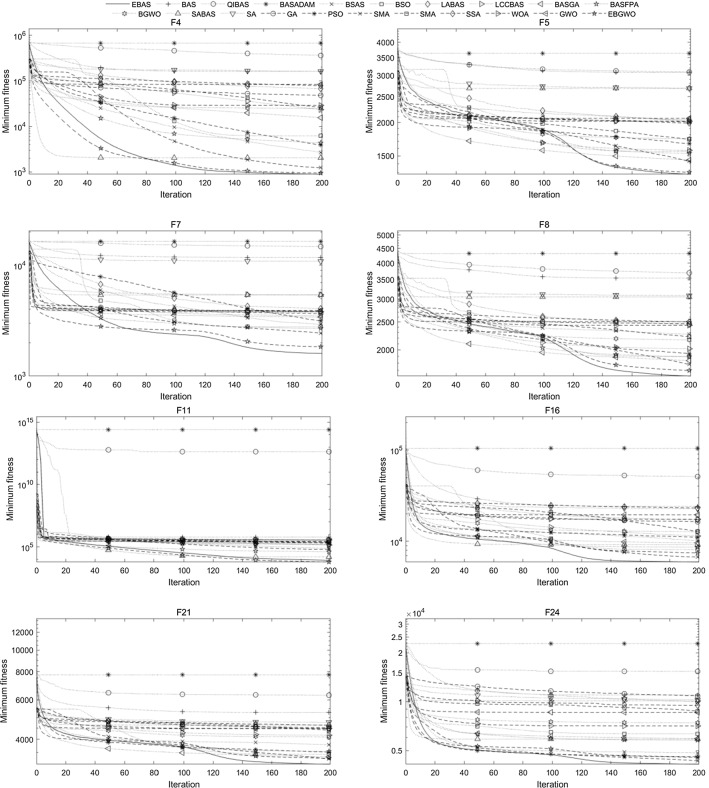
Convergence curve of partial functions for 100-dimensional problems

It can be seen from the convergence curves (Figs. 3 and 4) that in most cases, the optimal values found by EBAS decrease smoothly. In addition, a trend can be perceived from the convergence curve of EBAS: The searching process of it is clearly divided into two stages. Specifically, there are rapid reductions in fitness values during both the early and the late period of iterations. This trend probably comes from the combination of the curve adaptive step size method and the contemporary optimal update strategy, by which EBAS focuses on global optimization in the early stage, thus locating the area around the global optimal solutions; then, EBAS focuses on local optimization at the later stage, so as to perform fine search near the current optimal solutions. In other words, EBAS gets a good balance between the exploration and the exploitation abilities. Furthermore, both the abilities are promoted with the aid of the multi-directional sensing method, resulting in better results than other algorithms.

As for the other comparative algorithms, EBGWO and SMA can also get relatively good results. SMA is a representational state-of-the-art algorithm, which has good searching ability. Among the improved algorithms of BAS, BAS_ADAM has very poor optimization ability, probably because the shortcomings mentioned in Sect. 1.2. The result of QIBAS is also not well. In QIBAS, the quadratic interpolation is used to calculate an interpolation solution based on the left and right solutions. Then, it is compared with the next solution by formula (2) to select the best one as the initial solution of the next iteration. Since the present study utilized complex test functions, quadratic interpolation may not be able to approximate the tendency of the functions, thus resulting in bad performance.

### Statistic analysis

The Wilcoxon rank sum test (Alcalá-Fdez et al. 2009) is a nonparametric method for testing significant differences in results, which can be used for pairwise comparisons of algorithms. The comparison result is the p value. When the p value is less than 0.05, it indicates that the optimization results of the two algorithms are statistically different; and when the p value is greater than 0.05, it is considered that the results are not statistically different. The above experimental data based on 29 test functions are used to judge whether the proposed EBAS is significantly different from the 20 comparison algorithms. Table 15 shows that the results of EBAS are significantly better than other algorithms in most of the test functions, both in 50-dimensional and 100-dimensional problems.

**Table 15:** The results of the Wilcoxon rank sum test

	*D* = 50			*D* = 100		
	+	=	−	+	=	−
BAS	29	0	0	29	0	0
QIBAS	27	1	1	27	1	1
BAS_ADAM	29	0	0	29	0	0
BSAS	28	1	0	29	0	0
BSO	24	2	3	27	1	1
LABAS	28	0	1	28	0	1
LCCBAS	27	2	0	29	0	0
BASGA	25	3	1	25	3	1
BASFPA	23	3	3	24	2	3
BGWO	26	3	0	28	0	1
SABAS	19	2	8	20	2	7
SA	29	0	0	29	0	0
GA	29	0	0	29	0	0
PSO	24	1	4	26	1	2
SMA	19	5	5	22	4	3
HHO	27	1	1	28	0	1
SSA	29	0	0	28	0	1
WOA	29	0	0	29	0	0
GWO	28	1	0	28	0	1
EBGWO	18	6	5	15	7	7
Overall	517	31	32	529	21	30
Average	25.85	1.55	1.6	26.45	1.1	1.5

‘ + ’ indicates the number of test functions in which EBAS are significantly better than the algorithm, ‘ = ’ indicates no significant difference, and ‘-’ indicates that EBAS’s optimization results are significantly worse than the algorithm

The Wilcoxon rank sum test can be used for the comparison between two algorithms, but it cannot evaluate the goodness of multiple algorithms. Therefore, the Friedman tests (Sheskin 2004) are further used to evaluate the performance of EBAS among comparison algorithms.

The ranking obtained by Friedman test is shown in Table 16. As we can see, EBAS is the best among the algorithms both in 50-dimensional and 100-dimensional problems. The rankings of EBGWO and SMA are relatively high besides EBAS, and the relatively high-ranking algorithms among the existing improved BAS algorithms are BASFPA and BSO. In addition, EBAS is superior to BSAS and BGA, which utilize multi-directional sensing method. For the data of 50-dimensional and 100-dimensional problems, the p values of Friedman test are both 0, indicating that the optimization results of 21 algorithms are significantly different. Further post hoc multiple comparisons are conducted and the results are shown in Table 17. The results of EBAS are significantly different from all other comparison algorithms.

**Table 16:** The ranking obtained by Friedman test

*N* = 50	EBAS	bas	QIBAS	BAS_ADAM	BSAS	BSO	LABAS	LCCBAS	BASGA	BASFPA
	1	18	19	21	11	5	16	9	7	4
BGWO	SABAS	SA	GA	PSO	SMA	HHO	SSA	WOA	GWO	EBGWO
10	8	20	14	6	3	12	15	13	17	2

N = 100	EBAS	bas	QIBAS	BAS_ADAM	BSAS	BSO	LABAS	LCCBAS	BASGA	BASFPA
	1	18	20	21	9	5	15	10	7	4
BGWO	SABAS	SA	GA	PSO	SMA	HHO	SSA	WOA	GWO	EBGWO
11	6	19	14	8	3	12	16	13	17	2

**Table 17:** The results of post hoc multiple comparisons test. ‘TRUE’ means the results are significantly different

	*D* = 50	*D* = 100
BAS VS EBAS	TRUE	TRUE
QIBAS VS EBAS	TRUE	TRUE
BAS_ADAM VS EBAS	TRUE	TRUE
BSAS VS EBAS	TRUE	TRUE
BSO VS EBAS	TRUE	TRUE
LABAS VS EBAS	TRUE	TRUE
LCCBAS VS EBAS	TRUE	TRUE
BASGA VS EBAS	TRUE	TRUE
BASFPA VS EBAS	TRUE	TRUE
BGWO VS EBAS	TRUE	TRUE
SABAS VS EBAS	TRUE	TRUE
SA VS EBAS	TRUE	TRUE
GA VS EBAS	TRUE	TRUE
PSO VS EBAS	TRUE	TRUE
SMA VS EBAS	TRUE	TRUE
HHO VS EBAS	TRUE	TRUE
SSA VS EBAS	TRUE	TRUE
WOA VS EBAS	TRUE	TRUE
GWO VS EBAS	TRUE	TRUE
EBGWO VS EBAS	TRUE	TRUE

### Additional test on CEC’15

In order to further show the performance of EBAS, additional tests are conducted based on the test functions of CEC’15. The name of basic functions in CEC’15 can be seen from Table 18. Although several basic functions in CEC’15 are the same as that in CEC’17, the hybrid and composition functions are not the same between the two test suites, making this additional test valuable. The basic functions of CEC’15 are also shifted, rotated, or combined to form the formal 15 test functions (among them 10 functions are hybrid or composition functions), which means the test functions are also unbiased. The procedure of the test is similar to the above experiments, the dimension of functions is set to 100 and the number of random directions for EBAS and EBGWO is set to 60.

**Table 18:** The number and name of basic functions in CEC’15

No	Name	No	Name
$f_{1}$	High Conditioned Elliptic	$f_{8}$	Rastrigin
$f_{{2}}$	Cigar	$f_{9}$	Modified Schwefel
$f_{{3}}$	Discus	$f_{10}$	Katsuura
$f_{{4}}$	Rosenbrock	$f_{11}$	HappyCat
$f_{{5}}$	Ackley	$f_{12}$	HGBat
$f_{{6}}$	Weierstrass	$f_{13}$	Expanded Griewank’s plus Rosenbrock
$f_{7}$	Griewank	$f_{14}$	Expanded Schaffer’s F6

The detailed test results can be found in Table 19, EBAS achieved the best results among all algorithms in F7 and F13. In addition, EBAS got the minimum Fbest and Fmean values in F4, F5, F11, and F12 and got the minimum Fbest values in F9 and F15.

**Table 19:** The comparative results for 100-dimensional problems from CEC’15

		EBAS	BAS	QIBAS	BAS_ADAM	BSAS	BSO	LABAS	LCCBAS	BASGA	BASFPA	BGWO	SABAS
F1	Fbest	6.1E + 07	3.3E + 09	8.4E + 09	2.9E + 10	3.4E + 08	3.9E + 08	3.0E + 09	5.2E + 08	6.4E + 08	1.7E + 08	1.1E + 09	**3.3E + 07**
	Fmean	1.1E + 08	9.4E + 09	3.2E + 10	6.0E + 10	6.9E + 08	6.5E + 08	5.5E + 09	1.1E + 09	1.1E + 09	4.3E + 08	1.8E + 09	**4.8E + 07**
	Std	2.6E + 07	2.5E + 09	1.3E + 10	2.5E + 10	1.9E + 08	1.5E + 08	1.1E + 09	4.0E + 08	2.7E + 08	1.3E + 08	3.7E + 08	**1.1E + 07**
F2	Fbest	1.9E + 07	3.2E + 11	4.9E + 11	6.1E + 11	1.1E + 10	3.5E + 10	2.3E + 11	1.3E + 11	9.2E + 10	2.0E + 10	1.2E + 11	**2.0E + 02**
	Fmean	3.0E + 07	4.7E + 11	7.2E + 11	9.0E + 11	2.7E + 10	5.2E + 10	2.8E + 11	1.5E + 11	1.3E + 11	3.2E + 10	1.6E + 11	**8.1E + 03**
	Std	1.1E + 07	5.4E + 10	1.4E + 11	1.3E + 11	7.0E + 09	8.4E + 09	1.9E + 10	1.8E + 10	1.7E + 10	8.1E + 09	1.8E + 10	**8.1E + 03**
F3	Fbest	3.2E + 02	3.2E + 02	3.2E + 02	3.2E + 02	3.2E + 02	3.2E + 02	3.2E + 02	3.2E + 02	3.2E + 02	3.2E + 02	3.2E + 02	**3.2E + 02**
	Fmean	3.2E + 02	3.2E + 02	3.2E + 02	3.2E + 02	3.2E + 02	3.2E + 02	3.2E + 02	3.2E + 02	3.2E + 02	3.2E + 02	3.2E + 02	**3.2E + 02**
	Std	3.3E-02	6.1E-02	2.9E-01	2.6E-02	3.2E-02	7.7E-02	2.1E-02	6.8E-02	9.6E-02	1.5E-01	8.6E-02	**6.2E−03**
F4	Fbest	**9.8E + 02**	2.4E + 03	2.5E + 03	3.3E + 03	1.6E + 03	1.4E + 03	1.9E + 03	1.3E + 03	1.3E + 03	1.2E + 03	1.6E + 03	1.9E + 03
	Fmean	**1.2E + 03**	3.0E + 03	3.0E + 03	3.8E + 03	1.9E + 03	1.5E + 03	2.0E + 03	1.6E + 03	1.4E + 03	1.5E + 03	1.7E + 03	2.7E + 03
	Std	1.2E + 02	2.9E + 02	2.8E + 02	2.8E + 02	1.6E + 02	8.1E + 01	7.2E + 01	1.3E + 02	7.0E + 01	1.6E + 02	6.7E + 01	3.4E + 02
F5	Fbest	**1.3E + 04**	2.7E + 04	2.5E + 04	3.6E + 04	2.8E + 04	2.6E + 04	3.0E + 04	1.4E + 04	1.8E + 04	1.6E + 04	2.2E + 04	1.4E + 04
	Fmean	**1.5E + 04**	3.0E + 04	3.0E + 04	3.8E + 04	3.0E + 04	3.1E + 04	3.2E + 04	1.9E + 04	2.0E + 04	2.0E + 04	2.5E + 04	1.7E + 04
	Std	1.4E + 03	1.6E + 03	2.1E + 03	1.6E + 03	1.6E + 03	1.7E + 03	7.4E + 02	4.7E + 03	1.4E + 03	1.8E + 03	1.7E + 03	2.0E + 03
F6	Fbest	4.7E + 06	3.4E + 08	1.3E + 09	3.8E + 09	3.2E + 07	2.2E + 07	3.0E + 08	2.3E + 07	2.2E + 07	1.0E + 07	4.5E + 07	**1.3E + 06**
	Fmean	9.5E + 06	1.0E + 09	9.2E + 09	2.4E + 10	5.0E + 07	4.3E + 07	8.8E + 08	1.3E + 08	8.2E + 07	2.5E + 07	1.8E + 08	**4.7E + 06**
	Std	2.6E + 06	5.5E + 08	7.4E + 09	1.1E + 10	1.5E + 07	1.3E + 07	2.7E + 08	2.7E + 08	4.1E + 07	1.0E + 07	8.0E + 07	**1.6E + 06**
F7	Fbest	**8.2E + 02**	3.5E + 03	1.1E + 04	3.8E + 04	9.1E + 02	1.1E + 03	2.9E + 03	1.4E + 03	1.2E + 03	9.9E + 02	1.5E + 03	8.4E + 02
	Fmean	**8.6E + 02**	9.5E + 03	3.5E + 04	9.0E + 04	1.0E + 03	1.3E + 03	5.1E + 03	1.8E + 03	1.5E + 03	1.1E + 03	2.0E + 03	1.4E + 03
	Std	**2.1E + 01**	4.0E + 03	2.5E + 04	3.8E + 04	5.7E + 01	8.9E + 01	1.0E + 03	3.0E + 02	1.4E + 02	6.9E + 01	2.8E + 02	6.5E + 02
F8	Fbest	1.1E + 06	1.2E + 08	5.0E + 08	1.5E + 09	1.2E + 07	5.6E + 06	1.3E + 08	6.3E + 06	1.5E + 07	3.2E + 06	3.2E + 07	**6.5E + 05**
	Fmean	5.7E + 06	4.6E + 08	3.0E + 09	9.3E + 09	3.1E + 07	1.7E + 07	2.2E + 08	2.0E + 07	3.9E + 07	1.1E + 07	7.5E + 07	**2.8E + 06**
	Std	2.5E + 06	2.9E + 08	3.1E + 09	4.9E + 09	1.2E + 07	7.0E + 06	6.7E + 07	1.2E + 07	1.9E + 07	4.5E + 06	3.0E + 07	**1.2E + 06**
F9	Fbest	**1.0E + 03**	3.1E + 03	2.7E + 03	5.0E + 03	1.0E + 03	1.1E + 03	2.3E + 03	1.7E + 03	1.3E + 03	1.1E + 03	1.6E + 03	1.0E + 03
	Fmean	1.3E + 03	5.6E + 03	7.6E + 03	1.7E + 04	1.3E + 03	1.3E + 03	3.3E + 03	2.5E + 03	1.6E + 03	1.4E + 03	2.3E + 03	1.9E + 03
	Std	3.8E + 02	2.8E + 03	3.1E + 03	6.3E + 03	4.8E + 02	**1.8E + 02**	5.7E + 02	7.7E + 02	2.7E + 02	4.7E + 02	6.8E + 02	1.1E + 03
F10	Fbest	2.6E + 06	3.3E + 08	4.1E + 08	4.9E + 09	1.2E + 07	7.1E + 06	3.0E + 08	6.4E + 06	4.5E + 07	4.3E + 06	5.2E + 07	**2.0E + 06**
	Fmean	7.4E + 06	9.1E + 08	6.8E + 09	2.2E + 10	4.0E + 07	2.1E + 07	6.9E + 08	4.1E + 07	1.4E + 08	1.3E + 07	2.3E + 08	**3.9E + 06**
	Std	3.4E + 06	5.0E + 08	5.6E + 09	1.2E + 10	1.5E + 07	1.1E + 07	2.1E + 08	3.4E + 07	5.7E + 07	5.7E + 06	1.3E + 08	**1.4E + 06**
F11	Fbest	**1.5E + 03**	6.1E + 03	6.5E + 03	1.3E + 04	1.9E + 03	5.2E + 03	5.6E + 03	6.2E + 03	3.2E + 03	2.2E + 03	4.6E + 03	1.6E + 03
	Fmean	**3.4E + 03**	7.3E + 03	1.7E + 04	3.5E + 04	4.0E + 03	5.6E + 03	6.6E + 03	7.4E + 03	5.3E + 03	5.1E + 03	5.8E + 03	5.0E + 03
	Std	1.1E + 03	7.9E + 02	1.3E + 04	1.8E + 04	1.2E + 03	2.0E + 02	3.6E + 02	8.7E + 02	4.7E + 02	6.2E + 02	3.4E + 02	1.3E + 03
F12	Fbest	**1.3E + 03**	1.5E + 03	1.4E + 03	1.7E + 03	1.3E + 03	1.4E + 03	1.4E + 03	1.4E + 03	1.4E + 03	1.4E + 03	1.4E + 03	1.3E + 03
	Fmean	**1.4E + 03**	1.5E + 03	1.7E + 03	2.8E + 03	1.4E + 03	1.4E + 03	1.4E + 03	1.5E + 03	1.4E + 03	1.4E + 03	1.5E + 03	1.4E + 03
	Std	4.0E + 01	5.4E + 01	3.1E + 02	7.9E + 02	3.2E + 01	7.1E + 00	1.8E + 01	2.6E + 01	1.2E + 01	1.2E + 01	3.2E + 01	3.9E + 01
F13	Fbest	**1.3E + 03**	1.6E + 03	1.3E + 03	1.7E + 04	1.3E + 03	1.3E + 03	1.5E + 03	1.5E + 03	1.3E + 03	1.3E + 03	1.3E + 03	1.3E + 03
	Fmean	**1.3E + 03**	7.2E + 03	2.1E + 04	5.3E + 04	1.3E + 03	1.3E + 03	3.8E + 03	3.2E + 03	1.3E + 03	1.3E + 03	1.5E + 03	5.0E + 03
	Std	**7.9E−02**	8.6E + 03	1.6E + 04	1.9E + 04	1.9E-01	1.5E + 01	1.4E + 03	1.6E + 03	1.9E + 01	9.9E + 00	1.3E + 02	1.1E + 04
F14	Fbest	1.3E + 05	4.4E + 05	6.1E + 05	5.5E + 06	1.4E + 05	2.1E + 05	3.9E + 05	3.6E + 05	2.4E + 05	1.6E + 05	3.1E + 05	1.2E + 05
	Fmean	1.5E + 05	9.0E + 05	5.4E + 06	4.1E + 07	1.6E + 05	3.2E + 05	5.4E + 05	5.9E + 05	3.5E + 05	2.0E + 05	6.7E + 05	1.5E + 05
	Std	1.2E + 04	3.4E + 05	1.2E + 07	5.5E + 07	1.1E + 04	5.8E + 04	7.9E + 04	1.3E + 05	1.0E + 05	1.9E + 04	1.4E + 05	1.5E + 04
F15	Fbest	**1.6E + 03**	5.6E + 05	1.3E + 06	7.2E + 06	1.7E + 03	4.3E + 03	1.3E + 05	4.1E + 03	1.5E + 04	2.6E + 03	3.2E + 04	1.6E + 03
	Fmean	1.6E + 03	2.2E + 06	1.1E + 07	5.9E + 07	2.6E + 03	9.0E + 03	2.3E + 05	3.0E + 04	2.5E + 04	4.4E + 03	5.2E + 04	2.2E + 03
	Std	6.9E + 00	1.3E + 06	8.0E + 06	5.1E + 07	1.0E + 03	2.6E + 03	5.7E + 04	1.4E + 04	7.5E + 03	1.7E + 03	1.1E + 04	9.4E + 02

		SA	GA	PSO	SMA	HHO	SSA	WOA	GWO	EBGWO
F1	Fbest	9.0E + 09	2.2E + 09	5.3E + 08	1.2E + 08	8.4E + 08	3.2E + 09	1.7E + 09	4.3E + 09	3.7E + 07
	Fmean	1.2E + 10	3.5E + 09	1.0E + 09	2.2E + 08	1.4E + 09	5.9E + 09	2.5E + 09	6.6E + 09	1.3E + 08
	Std	1.2E + 09	5.0E + 08	2.8E + 08	5.3E + 07	3.3E + 08	1.1E + 09	5.2E + 08	1.2E + 09	4.3E + 07
F2	Fbest	3.7E + 11	2.3E + 11	1.7E + 10	1.2E + 09	1.1E + 11	2.2E + 11	1.2E + 11	2.5E + 11	2.0E + 07
	Fmean	4.7E + 11	2.6E + 11	2.4E + 10	2.4E + 09	1.5E + 11	2.9E + 11	1.7E + 11	3.0E + 11	2.6E + 07
	Std	3.0E + 10	1.7E + 10	4.4E + 09	5.5E + 08	1.4E + 10	2.5E + 10	1.5E + 10	1.8E + 10	4.3E + 06
F3	Fbest	3.2E + 02	3.2E + 02	3.2E + 02	3.2E + 02	3.2E + 02	3.2E + 02	3.2E + 02	3.2E + 02	3.2E + 02
	Fmean	3.2E + 02	3.2E + 02	3.2E + 02	3.2E + 02	3.2E + 02	3.2E + 02	3.2E + 02	3.2E + 02	3.2E + 02
	Std	4.9E-02	2.9E-02	3.6E-02	3.0E-02	4.8E-02	1.7E-01	5.9E-02	3.0E-02	5.2E-02
F4	Fbest	2.4E + 03	1.9E + 03	1.4E + 03	1.2E + 03	1.6E + 03	1.9E + 03	1.9E + 03	1.9E + 03	1.2E + 03
	Fmean	2.7E + 03	2.0E + 03	1.5E + 03	1.4E + 03	1.7E + 03	2.0E + 03	2.0E + 03	2.0E + 03	1.3E + 03
	Std	6.6E + 01	5.9E + 01	8.7E + 01	8.9E + 01	6.8E + 01	**5.3E + 01**	9.8E + 01	6.4E + 01	7.1E + 01
F5	Fbest	3.1E + 04	2.9E + 04	3.0E + 04	1.7E + 04	2.0E + 04	2.3E + 04	2.6E + 04	2.9E + 04	1.3E + 04
	Fmean	3.2E + 04	3.2E + 04	3.2E + 04	2.0E + 04	2.5E + 04	2.6E + 04	3.0E + 04	3.2E + 04	1.6E + 04
	Std	**6.8E + 02**	8.7E + 02	1.0E + 03	1.5E + 03	1.9E + 03	1.5E + 03	1.7E + 03	9.6E + 02	1.5E + 03
F6	Fbest	8.9E + 08	1.9E + 08	5.2E + 07	1.9E + 07	8.7E + 07	2.7E + 08	1.2E + 08	4.5E + 08	4.5E + 06
	Fmean	1.5E + 09	3.8E + 08	1.0E + 08	4.6E + 07	2.8E + 08	8.6E + 08	3.4E + 08	1.2E + 09	1.0E + 07
	Std	2.7E + 08	8.7E + 07	3.5E + 07	1.9E + 07	1.6E + 08	4.0E + 08	1.5E + 08	5.3E + 08	3.4E + 06
F7	Fbest	6.4E + 03	2.7E + 03	9.6E + 02	8.5E + 02	1.6E + 03	3.5E + 03	1.7E + 03	4.1E + 03	8.3E + 02
	Fmean	1.0E + 04	3.4E + 03	1.1E + 03	8.9E + 02	2.2E + 03	5.9E + 03	2.4E + 03	6.7E + 03	8.8E + 02
	Std	1.4E + 03	4.9E + 02	6.6E + 01	2.8E + 01	3.4E + 02	1.6E + 03	4.0E + 02	1.8E + 03	2.4E + 01
F8	Fbest	2.4E + 08	6.5E + 07	1.7E + 07	8.6E + 06	3.4E + 07	6.2E + 07	4.4E + 07	1.6E + 08	2.9E + 06
	Fmean	5.4E + 08	1.5E + 08	4.7E + 07	2.6E + 07	6.9E + 07	2.2E + 08	9.6E + 07	3.7E + 08	6.8E + 06
	Std	1.2E + 08	4.7E + 07	1.9E + 07	9.6E + 06	2.5E + 07	1.0E + 08	3.8E + 07	1.6E + 08	2.9E + 06
F9	Fbest	3.0E + 03	2.2E + 03	1.1E + 03	1.0E + 03	1.9E + 03	3.0E + 03	1.6E + 03	2.7E + 03	1.0E + 03
	Fmean	3.5E + 03	2.7E + 03	1.2E + 03	**1.1E + 03**	3.0E + 03	4.0E + 03	2.0E + 03	3.3E + 03	1.1E + 03
	Std	2.2E + 02	3.6E + 02	2.1E + 02	2.0E + 02	6.2E + 02	6.5E + 02	5.9E + 02	4.0E + 02	2.9E + 02
F10	Fbest	9.8E + 08	2.8E + 08	7.4E + 06	3.0E + 06	2.6E + 07	1.4E + 08	8.5E + 07	3.7E + 08	2.7E + 06
	Fmean	1.4E + 09	4.9E + 08	3.5E + 07	2.5E + 07	2.1E + 08	6.9E + 08	3.4E + 08	9.3E + 08	6.6E + 06
	Std	2.4E + 08	1.2E + 08	2.0E + 07	1.3E + 07	1.1E + 08	3.0E + 08	1.6E + 08	3.2E + 08	2.5E + 06
F11	Fbest	6.3E + 03	4.5E + 03	4.1E + 03	4.1E + 03	5.6E + 03	6.1E + 03	5.5E + 03	5.8E + 03	4.3E + 03
	Fmean	7.1E + 03	5.8E + 03	4.6E + 03	4.4E + 03	6.1E + 03	7.0E + 03	6.0E + 03	6.5E + 03	4.7E + 03
	Std	3.8E + 02	5.0E + 02	2.2E + 02	**1.7E + 02**	2.3E + 02	4.2E + 02	2.4E + 02	5.6E + 02	2.7E + 02
F12	Fbest	1.5E + 03	1.5E + 03	1.3E + 03	1.4E + 03	1.4E + 03	1.4E + 03	1.4E + 03	1.4E + 03	1.4E + 03
	Fmean	1.6E + 03	1.6E + 03	1.4E + 03	1.4E + 03	1.5E + 03	1.4E + 03	1.4E + 03	1.5E + 03	1.4E + 03
	Std	2.2E + 01	4.5E + 01	2.1E + 01	**3.7E-01**	2.4E + 01	2.7E + 01	2.7E + 00	1.9E + 01	2.9E + 01
F13	Fbest	3.0E + 03	1.8E + 03	1.3E + 03	1.3E + 03	1.3E + 03	1.4E + 03	1.3E + 03	1.3E + 03	1.3E + 03
	Fmean	4.0E + 03	2.2E + 03	1.3E + 03	1.3E + 03	1.7E + 03	3.1E + 03	1.3E + 03	1.7E + 03	1.3E + 03
	Std	5.4E + 02	4.0E + 02	6.1E-01	3.6E-01	2.4E + 02	2.3E + 03	7.0E + 00	3.3E + 02	4.6E + 00
F14	Fbest	5.6E + 05	5.0E + 05	1.7E + 05	**1.2E + 05**	3.4E + 05	3.4E + 05	2.5E + 05	4.5E + 05	1.3E + 05
	Fmean	9.0E + 05	7.9E + 05	1.8E + 05	**1.4E + 05**	5.5E + 05	5.1E + 05	3.2E + 05	7.1E + 05	2.8E + 05
	Std	1.3E + 05	1.5E + 05	**7.9E + 03**	1.2E + 04	1.1E + 05	1.2E + 05	5.4E + 04	8.1E + 04	2.1E + 05
F15	Fbest	1.6E + 06	7.1E + 04	2.3E + 03	1.6E + 03	1.4E + 04	1.2E + 05	4.0E + 04	2.4E + 05	1.6E + 03
	Fmean	2.6E + 06	1.3E + 05	5.7E + 03	1.7E + 03	3.1E + 04	2.2E + 05	6.5E + 04	3.3E + 05	**1.6E + 03**
	Std	4.5E + 05	3.0E + 04	3.1E + 03	6.2E + 01	8.2E + 03	4.7E + 04	1.8E + 04	5.9E + 04	**5.4E + 00**

Table 20 shows the results of the statistical analysis and the running times. As we can see, the results of EBAS are significantly better than other algorithms in most of the test functions by Wilcoxon rank sum test, and the ranking by Friedman test of EBAS is also the best. The post hoc multiple comparisons confirmed that the EBAS is significantly different from most of the algorithms. Besides EBAS, EBGWO and SMA also show good performance. SABAS and BASFPA have good searching ability among the improved BAS algorithms, though they are not as good as EBAS. EBAS outperformed BSAS and BGA, which utilize multi-directional sensing method. Similar to the previous analysis, BAS_ADAM and QIBAS have very poor optimization ability.

**Table 20:** The results of the Wilcoxon rank sum test between EBAS and other algorithms, Ranking by Friedman test, post hoc multiple comparisons tests between EBAS and other algorithms, and the average running time of each algorithm

	Wilcoxon			Ranking	Post hoc	Running Time
	+	=	−			
EBAS	–	–	–	1	–	3.2
BAS	15	0	0	18	TRUE	0.1
QIBAS	15	0	0	20	TRUE	0.1
BAS_ADAM	15	0	0	21	TRUE	0.4
BSAS	14	1	0	6	TRUE	1.2
BSO	15	0	0	8	TRUE	2.9
LABAS	15	0	0	16	TRUE	3.3
LCCBAS	15	0	0	10	TRUE	7.8
BASGA	15	0	0	9	TRUE	29.6
BASFPA	15	0	0	5	FALSE	4.1
BGWO	15	0	0	11	TRUE	1.0
SABAS	7	2	6	4	FALSE	5.4
SA	15	0	0	19	TRUE	3.7
GA	15	0	0	15	TRUE	2.8
PSO	15	0	0	7	TRUE	1.0
SMA	14	0	1	3	FALSE	2.3
HHO	15	0	0	13	TRUE	2.2
SSA	15	0	0	14	TRUE	1.4
WOA	15	0	0	12	TRUE	1.0
GWO	15	0	0	17	TRUE	0.9
EBGWO	6	7	1	2	FALSE	4.1
Overall	281	10	8	–	–	–
Average	14.05	0.5	0.4	–	–	–

### Additional tests on WSN coverage application problems

The previous experiments have shown the efficiency of EBAS on benchmark functions of CEC’17 and CEC’15. Although the functions are designed to simulate the real-world complex optimization problems, the global problem of related studies is the lack of validation on real-world engineering problems. Therefore, the performance of EBAS is further tested on the node coverage problem of Wireless Sensor Networks (WSN).

A WSN is a network in which multiple sensors are connected to each other for information transmission (Attea et al. 2019). With the continuous development of wireless sensor network, it has been widely used in rail transportation, medical equipment positioning, vehicle tracking and other fields. The primary problem of building a complete wireless network is to improve the monitoring range of sensors in the network. In practical applications, sensor nodes are deployed randomly in most cases, resulting in low node coverage. In this section, EBAS is used to find the optimal node deployment based on the Boolean perception model (Formula 10).

Assuming that the sensor network is arranged in a three-dimensional space V, there are n points to be detected (usually evenly distributed around the space), and m sensors are placed in the space. The coverage area of sensor nodes is a spherical area with itself as the center and r as the radius. All wireless sensor nodes in the area are (*N*1, *N*2, *N*3…*Ni*…*Nm*), all target detection points are (*P*1, *P*2, *P*3…*P*j…*Pn*), *d*(*Ni*, *Pj*) is the Euclidean distance between sensor node *Ni* and monitor point *Pj*. In this paper, the three-dimensional space *V* is discretized into a space composed of *K* points, which means the number of need to be detected points *n* = *K*. If the distance from a point in the space to the sensor is less than R, the point is considered to be covered. The probability model is as follows:

10 $$ P{(}Ni{,}Pj{)} = \left\{ \begin{gathered} {1, }d{(}Ni{,}Pj{) } \le \, r \hfill \\ {\text{0, else }} \hfill \\ \end{gathered} \right. $$

In formula (10), *P(Ni*, *Pj*) represents the probability that the sensor *Ni* perceives the monitoring point *Pj*. In this space *V*, any target point can be covered by multiple sensor nodes at the same time, and the joint coverage probability is

11 $$ P{(}N{,}Pj{)} = 1 - \prod {_{i = 1}^{m} } [1 - P(Ni,Pj)] $$

The network coverage rate *Cr* is defined as the ratio of the number of points covered to the total number of points *K* in the space:

12 $$ Cr = \frac{{\sum {_{j = 1}^{n} } P(N,Pj)}}{K} $$

This formula is also the fitness function of the model, that is, to find the maximum value of *Cr*.

In the experiment, the size of the three-dimensional space *V* is set to 20 × 20 × 20, the perception radius R is 4 or 5, 30 sensors are placed in the space, resulting in a 3*30 = 90-dimensional problem. The number of random directions is set to 60 for EBAS and EBGWO, the population size is set to 30, max iteration number is 100, each algorithm runs 30 times, the other parameters of the algorithms are the same as that in Sect. 6.1.

As we can see from Table 21 that the solution found by EBAS or EBGWO (which utilized EBAS as an additional operation) has the best coverage rate. The results proved the validity of EBAS on real-world engineering problems. This result is not surprising, since the searching behavior of EBAS does not depend on the simple patterns of solution space, which are usually contained in the biased test functions. In real-world applications, we cannot rely on the biased assumption, such as optimal solution may close to the origin of the solution space, to get better solutions. The present results based on the unbiased test functions and the WSN coverage problem proved the value of EBAS in real-world engineering applications.

**Table 21:** The results of the WSN coverage problem. The data are the coverage rate (%) under different algorithms and parameter R conditions

	*R* = 4		*R* = 5	
	Fbest	Fmean	Fbest	Fmean
EBAS	**76.4**	**74.1**	97.7	**96.5**
BAS	64.8	59.4	88.8	83.4
QIBAS	65.7	60.0	89.1	83.9
BAS_ADAM	57.6	53.0	82.7	75.2
BSAS	68.5	66.8	93.7	91.5
BSO	70.0	66.5	93.0	89.8
LABAS	64.3	62.4	87.2	85.1
LCCBAS	73.5	68.4	95.7	92.3
BASGA	72.1	69.5	93.6	91.0
BASFPA	74.0	71.0	96.3	94.2
BGWO	69.8	67.3	94.1	90.6
SABAS	67.1	64.0	92.2	88.0
SA	60.3	55.7	83.7	77.5
GA	65.0	61.7	88.2	85.1
PSO	72.5	69.5	94.5	92.8
SMA	61.8	58.8	84.7	81.4
HHO	69.1	65.4	91.9	89.2
SSA	66.0	62.5	89.4	84.4
WOA	66.5	62.6	88.7	86.3
GWO	66.7	62.7	89.7	85.5
EBGWO	76.3	73.4	**97.9**	96.2

## Conclusion

This paper is aiming to solve the problem that the basic BAS algorithm has poor optimization effect in the optimization problem of complex and unbiased functions. First, the existing improved BAS algorithms are summarized, and the problems in these algorithms are discussed. Second, three improving methods of EBAS are proposed.

The first method is the adaptive step size reduction method based on the precision factor *A*, which can automatically calculate the step size of the specific iteration according to the maximum number of iterations. This method increases the ease of use of the algorithm. In addition, the curvilinearly reduction in step size can increase the global optimization ability in the early stage and perform fine local optimization at the later stage, so as to enhance the searching efficiency and speed up the convergence speed of the algorithm.

The second method is the contemporary optimal update strategy, that is, the optimal solution calculated on each iteration is used as the initial solution of the next iteration and to update the historical optimal solution. This strategy makes full use of the fitness information generated in each iteration, thus improving the algorithm optimization efficiency. Such strategy is also more in line with the real beetle foraging behavior. Indeed, a beetle will not jump to a far place unless the food near its antennas has been eaten up. The contemporary optimal update strategy works well with the curve step size reduction method to balance the global and local searching abilities of the algorithm, and to reduce the sensitivity of parameters in EBAS, thus increasing the robustness of the algorithm.

The third method is the multi-directional sensing method, which adopts multiple random directions on each iteration to calculate the left, the right, and the next solutions. This method is capable to fully explore the solution space near the current solution, thus increasing the possibility of finding a better solution. It also can perfectly cooperate with the contemporary optimal update strategy to promote the optimization efficiency. In addition, compared with the basic BAS, which has only one random direction, the multi-directional sensing method is also more in line with the behavior of the beetle in reality. This is because that the real beetle can easily change the orientation of its head or antennas to explore the surrounding environment. Although the multi-directional sensing method has been used by some existing research works, this paper conducts a more extensive analysis on the method. Specifically, two influential factors of the method are investigated: the similarity between random directions and the problem dimensions. As a result, the criterion to use the multi-directional sensing method is proposed. That is, using two random directions is already able to greatly improve the optimization efficiency of the algorithm, but it is not recommended to use too many random directions in low-dimensional problems. As for high-dimensional problems, the number of random directions can be increased, but it should not be too close to or exceeding the dimension of the problem. In addition, the running time of the algorithm can be adjusted by the number of random directions. If the running time is more important, the number of random directions can be reduced; if the optimization result is more important, the number of random directions can be increased.

Finally, based on the results of test functions from CEC’17 and CEC’15, EBAS is compared with basic BAS, various improved BAS algorithms, several classic algorithms, and several state-of-the-art intelligent algorithms. In addition to its authority, the advantage of using the test functions of CEC’17 and CEC’15 is the ability to avoid possible bias from algorithm design, which may make the algorithm to behave better when the global optimal solution is located at the origin of the solution space. The experimental results show that although only one beetle is utilized, EBAS has better optimization ability than the comparison algorithms, including the algorithms with a swarm of individuals. For example, EBAS outperformed BAS with at least 1 orders of magnitude difference. The performance of EBAS was even better than several state-of-the-art swarm-based algorithms, such as Slime Mold Algorithm and Grey Wolf Optimization, with similar running times. The flexibility of EBAS is also superior to other improvements of BAS, since the number of random directions can be easily adjusted. In addition, EBGWO, which also performs well, fully demonstrates the ease of use for EBAS to be easily integrated with other algorithms. Overall, EBAS outperformed a majority of compared intelligent algorithms in unbiased function optimization.

It is worth mentioning that SMA also obtains good results, indicating that SMA is indeed a new and excellent intelligent algorithm with strong optimization ability. Since the performance of EBAS is superior to that of SMA in the present experiments, EBAS is obviously a successful improvement algorithm of BAS. Furthermore, among the existing improved BAS algorithms, BASFPA and BSO have relatively good searching ability, while BASGA consumes the longest running time and BAS_ADAM has the poorest searching ability.

As described in the no-free-lunch theorem, there is no universal algorithm that can solve all problems. Therefore, many research works are still trying to propose new metaheuristic algorithms, including the present study. Several latest algorithms are as follows, hybrid harmony search algorithm with hill climbing (Al-Shaikh et al. 2021), chemical reaction optimization (Mahafzah et al. 2021), most valuable player algorithm (Khattab et al. 2019), and sea lion optimization (Masadeh et al. 2021), and so on. All these algorithms and the algorithms in the literature have been used to solve many optimization problems, such as social network contact racing of COVID-19, scheduling problem, and minimum vertex cover problem. The metaheuristic algorithms play an increasingly important role in social production and people’s daily lives.

In the future research, we plan to apply EBAS to more practical engineering problems, and explore possible methods to further improve the optimization efficiency of EBAS.

## Data Availability

The data that support the findings of this study are available from the corresponding author, upon reasonable request.
